# Obesity among adults living in post-earthquake container settlements: a cross-sectional study in Malatya, Türkiye

**DOI:** 10.1136/bmjopen-2026-122076

**Published:** 2026-09-23

**Authors:** Osman Kurt, Aleyna Aslan, Ahmet Burak Avcu, Ayşe Baran, Ali Özer, Metin Fikret Genç, Harun Kurt

**Affiliations:** 1 Public Health, Faculty of Medicine, Inonu University, Malatya, Türkiye; 2 Malatya Provincial Health Directorate, Battalgazi, Malatya, Türkiye

**Keywords:** Obesity, Body Mass Index, PUBLIC HEALTH

## Abstract

**Abstract:**

**Objectives:**

To estimate the prevalence of obesity and identify the factors independently associated with it among adults living in container settlements established after the 6 February 2023 Kahramanmaraş earthquakes.

**Design:**

Population-based, descriptive, cross-sectional field study using stratified random sampling.

**Setting:**

Eight container settlements in Malatya, Türkiye, with on-site primary health services. Fieldwork was conducted between June and October 2025, 24–32 months after the earthquakes.

**Participants:**

Participants were adults aged ≥18 years permanently residing in the settlements (source population, n=27 997). Of 682 individuals randomly selected from settlement resident lists, 62 could not be contacted and were replaced by further random draws; of the 620 adults then approached, 57 were excluded for non-eligibility, incomplete data or refusal, yielding a final analytic sample of 563 (84.0% of all eligible selected individuals; 94.6% of those contacted).

**Primary and secondary outcome measures:**

Primary outcome: obesity (body mass index (BMI) ≥30.0 kg/m²) from measured height and weight. Secondary outcomes: distribution of BMI categories and factors associated with obesity assessed by logistic regression and, as a sensitivity analysis, by modified Poisson regression yielding prevalence ratios.

**Results:**

Obesity prevalence was 28.8% (162/563; 95% CI 25.2 to 32.6), falling to 24.4% (21.1 to 27.9) after standardisation to an equal sex distribution. Prevalence was markedly higher in women than in men (35.5% vs 13.5%) and differed across settlements (18.8%–66.7%; χ²=29.21, p<0.001) although estimates for the three smallest sites were imprecise. Six factors were independently associated with obesity: female sex (adjusted OR (AOR) 3.46, 95% CI 1.86 to 6.44), age ≥55 years (AOR 2.44, 95% CI 1.17 to 5.09), fast eating (AOR 2.28, 95% CI 1.39 to 3.74), self-reported childhood overweight (AOR 1.93, 95% CI 1.21 to 3.07), chronic disease (AOR 1.85, 95% CI 1.17 to 2.94) and low household income (AOR 1.73, 95% CI 1.05 to 2.86); the income association was sensitive to model specification (Nagelkerke R²=0.267; apparent, in-sample discrimination AUC=0.775, 95% CI 0.732 to 0.818). Associations persisted when settlement was added as a fixed effect, when cluster-robust variance was used and when prevalence ratios were estimated instead of ORs, with the exception of household income, which lost significance in the settlement-adjusted model.

**Conclusions:**

Obesity is a substantial, identifiable problem during the sub-acute to chronic phase of post-earthquake displacement in the Eastern Mediterranean. The profile of associated factors supports targeted intervention through on-site primary care, with older women who have chronic disease as the clearest priority group; the association with low household income was sensitive to adjustment for settlement and should be treated as provisional. The variation between settlements indicates that settlement-level environments warrant direct measurement in future work.

**STRENGTHS AND LIMITATIONS OF THIS STUDY**

- Population-based stratified random sampling from the full adult population (n=27 997) of all eight Malatya container settlements, with 563 of the 670 eligible adults selected taking part (84.0%).
- Body mass index was derived from directly measured height and weight, not self-report.
- Fieldwork at 24–32 months after the disaster captures the sub-acute to chronic phase of post-earthquake displacement that previous post-disaster cohorts have largely skipped.
- The cross-sectional design precludes causal inference, no pre-disaster anthropometric baseline is available and settlement conditions were recorded from participant reports rather than by objective measurement of the built or food environment.
- Childhood weight, eating speed and dietary patterns were captured by self-report and are subject to recall and social-desirability bias; daytime fieldwork also left men less often contactable, so women are over-represented, and prevalence is reported both crude and standardised to an equal sex distribution.

## Introduction

 Obesity has emerged as one of the most pressing public health issues of the 21st century and, with the exception of certain regions in sub-Saharan Africa and South Asia, has surpassed malnutrition as a contributor to global mortality rates.^1^ In 2022, 890 million adults (approximately 16% of the global adult population) were living with obesity.^1^ A high body mass index (BMI) is a known cause of cardiovascular diseases, type 2 diabetes, obstructive sleep apnoea, osteoarthritis and various region-specific cancers, and the years of life lost due to high BMI have increased significantly over the last 30 years.^2^ Although the pathophysiology reflects a complex interplay of genetic, neuroendocrine and behavioural factors, contemporary frameworks emphasise that population-level patterns are increasingly shaped by upward-acting determinants: poverty, employment, urbanisation, food production and marketing and the wider built environment.^3 4^ It is therefore essential to identify the contexts where these determinants converge most intensely to design targeted prevention strategies.

Turkey is one of the countries with the highest burden of obesity in the European region. According to the WHO Regional Report on Obesity in Europe 2022, Turkey has the highest prevalence of obesity among adults in the WHO European Region, with 32.1% of adults classified as obese and 66.8% as overweight or obese.^5^ National figures from the 2022 Turkey Health Survey indicate an obesity rate of 20.2% (23.6% among women, 16.8% among men),^6^ and TURDEP-II analyses estimated adult obesity at 31.5% in 2022 and projected it to reach 34.9% by 2045.^7^ In this context, the prevalence of obesity is consistently higher among women with limited education and in households experiencing financial hardship, suggesting that environmental shocks restricting food choices or physical activity may exacerbate pre-existing inequalities.^8 9^

Natural disasters and the prolonged displacement that follows constitute one such environmental shock. A growing body of longitudinal evidence from cohorts established following the 2011 Great East Japan Earthquake has shown that residents housed in prefabricated temporary accommodation experienced clinically significant weight gain lasting for years. Hikichi *et al* reported that approximately 2.5 years after the disaster, the prevalence of obesity among displaced survivors rose from 25.0% to 35.1%, while it remained essentially unchanged in non-displaced control groups (from 26.9% to 26.6%).^10^ Subsequent 5-year and 7-year follow-ups of the Fukushima Health Management Study confirmed that this trend associated with temporary housing persisted well beyond the acute phase.^11–13^ A systematic review of 17 studies (229 058 participants) concluded that natural disasters consistently increase obesity, with displacement, restricted access to fresh food, sedentary lifestyle and chronic psychosocial stress being the primary mediating factors.^14^ Two significant limitations of this literature are noteworthy: the cohorts are concentrated in East Asia, and the Eastern Mediterranean region has largely not been characterised^15^; furthermore, the 18–36 month subacute-to-chronic transition period, during which displaced households move from short-term coping strategies to a settled new lifestyle, has rarely been examined as a distinct phase.^16^

On 6 February 2023, this fault line took on sudden and significant importance for Turkey. Two successive earthquakes, with moment magnitudes of 7.7 and 7.6 and epicentres in Pazarcık and Elbistan (Kahramanmaraş province), occurred 9 hours apart along the East Anatolian Fault, resulting in the deadliest earthquake in modern Turkish history. These earthquakes resulted in 53 537 confirmed deaths and 107 204 injuries across 11 provinces and a population of approximately 14 million, displacing an estimated nine million people.^17 18^ Malatya was among the hardest-hit provinces, where the seismic intensity reached Modified Mercalli Intensity (MMI) VIII–IX and tens of thousands of buildings became uninhabitable.^19^ As a medium-term housing solution, the Disaster and Emergency Management Presidency (AFAD) established 305 container housing sites across 10 provinces.^20^ Each prefabricated unit is approximately 21 m² in size and is typically used by four to six household members; most had moved from family homes well over 100 m². Two years after the disaster, over 200 000 people in Hatay province alone continued to live in container homes, and similar conditions persisted in Malatya; here, shared hygiene facilities, limited cooking infrastructure, restricted physical activity space and limited access to fresh produce remained defining features of daily life.^21 22^ This sudden and prolonged restriction of domestic space, dietary autonomy and routine activity represents a structural exposure with potential biological obesogenic consequences (figure 1); however, its quantitative impact on adult body weight in the post-disaster context in Turkey has not previously been measured.

**Figure 1:**
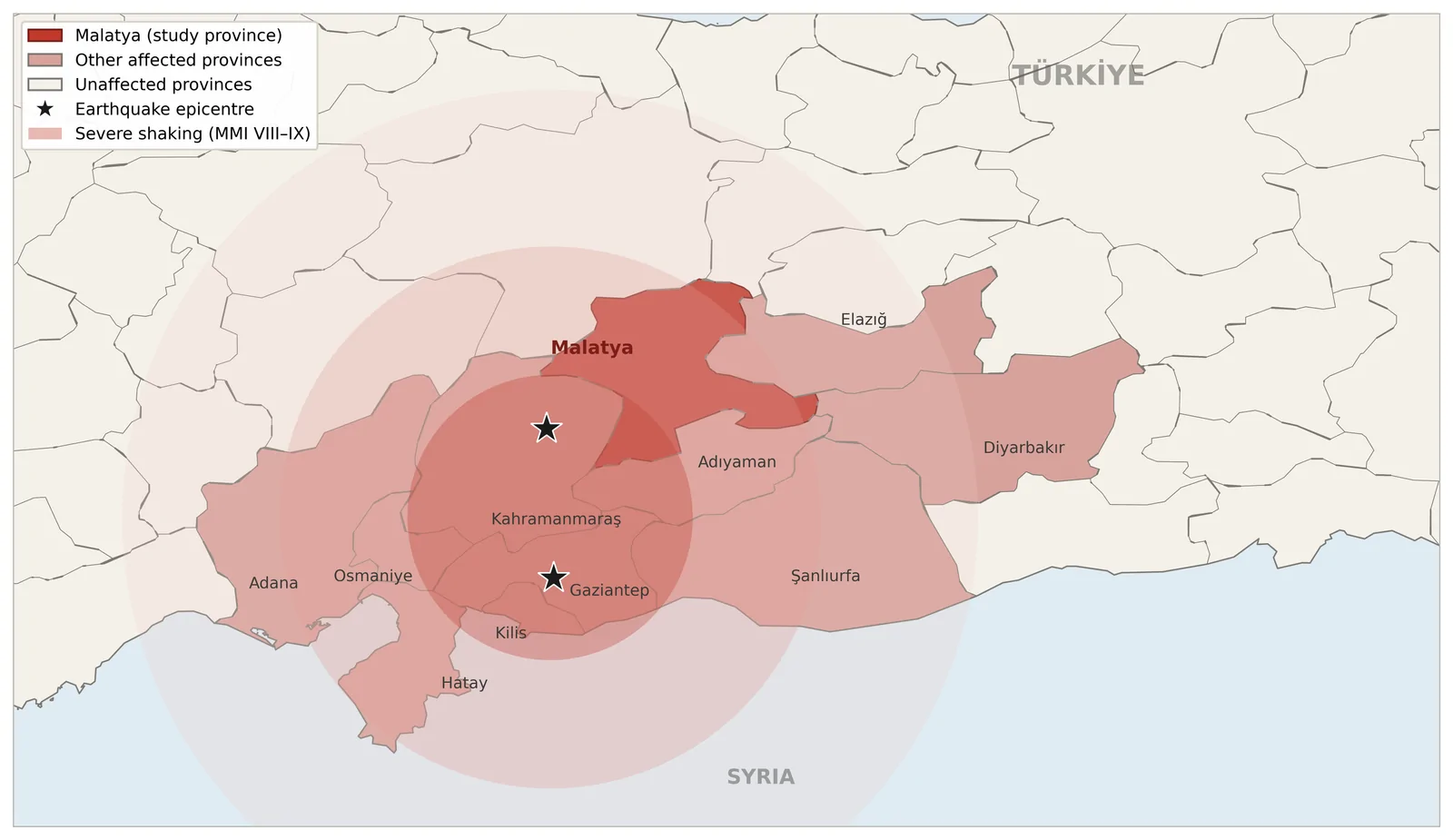
Study area. South-eastern Türkiye showing the 11 provinces affected by the 6 February 2023 Kahramanmaraş earthquakes, with Malatya (the study province) highlighted in dark red. Black stars mark the Mw 7.7 (Pazarcık, 04:17 local time) and Mw 7.6 (Elbistan, 13:24 local time) epicentres on the East Anatolian Fault Zone. Concentric red shading indicates approximate zones of severe shaking (MMI VIII–IX). MMI, Modified Mercalli Intensity; Mw, moment magnitude.

We therefore conducted a population-based cross-sectional study to estimate the prevalence of obesity among adults living in the eight container settlements established in Malatya following the earthquakes of 6 February 2023 and identify its independent sociodemographic, behavioural and clinical determinants. Data were collected 24–32 months after the disaster, positioning our study within the subacute to chronic phase of post-disaster displacement, a period that has been insufficiently researched. To our knowledge, this is the first population-based study on obesity among adults living in earthquake-related container settlements in the Eastern Mediterranean region. Findings will inform the planning of humanitarian aid and primary healthcare services for the ongoing recovery in Turkey and provide a transferable evidence base for similar post-disaster contexts where containerised or prefabricated medium-term housing has become the default response.

## Methods

### Study design and setting

This was a population-based, descriptive, cross-sectional field study. Data were collected from eight container settlements in Malatya province, established to house residents affected by the 6 February 2023 Kahramanmaraş earthquakes and where basic healthcare services are provided on-site: Battalgazi-TeknoKent, Battalgazi-Malet, Yeşilyurt-Manas, Yeşilyurt-MİAD, Yeşilyurt-Rönesans, Yeşilyurt-Mabek, Yeşilyurt-Yeni Hayat and Yeşilyurt-İkizce. The field study was conducted between June and October 2025.

### Population and sampling

The source population comprised adults aged ≥18 years residing permanently in the eight container settlements (total n=27 997). The required sample size was estimated using the finite-population formula n = [DEFF×N × p × (1−p)] / [(d² / Z²₁₋α/₂) × (N−1) + p × (1−p)], with a 95% confidence level, 5% margin of error, design effect (DEFF) of 2 and an adult obesity prevalence of 24.5% reported by the General Directorate of Public Health of Türkiye.^23^ These parameters yielded a minimum required sample of 563 individuals. A DEFF of 2 was applied as a conservative allowance for residual similarity between residents of the same settlement, a common choice in surveys of displaced populations where a single national sampling frame is unavailable. The eight settlements were treated as strata rather than as randomly sampled clusters, since every settlement in the province was included rather than a sample of them.

Participants were selected through a two-stage procedure. First, each container site was treated as an independent stratum, and the calculated sample was allocated in proportion to the resident population of each site: 114 in TeknoKent, 125 in Malet, 15 in Manas, 9 in MİAD, 54 in Rönesans, 181 in Mabek, 50 in Yeni Hayat and 15 in İkizce. Second, within each stratum, individuals rather than households were drawn. An up-to-date list of registered adult residents, obtained from the local settlement authority, served as the sampling frame, and named individuals were selected from it with an online random number generator before any field contact was made. No more than one adult per household was retained; because the overall sampling fraction was approximately 2%, two adults from the same household were seldom drawn, and this constraint was rarely binding. There was therefore no within-household selection step, selection probabilities were approximately equal across eligible adults and design weights were not required. Since the sample was allocated in proportion to the adult population of each settlement, the design is also approximately self-weighting across strata. A sensitivity analysis weighting participants by household size is reported in online supplemental table S1; because frequency weights do not reproduce design-based sampling variance, only point estimates are given there.

Eligible participants were adults aged ≥18 years who were permanent residents of one of the eight settlements and who gave their consent. Individuals who were pregnant or breastfeeding and had serious medical conditions preventing anthropometric measurement and communication difficulties were excluded.

Fieldwork took place during daytime hours. Of the 682 individuals selected, 62 could not be reached after repeated visits and were replaced by a further random draw from the same stratum; 51 of these 62 were men, reflecting the daytime absence of working-age men from the settlements. A total of 620 adults were therefore approached. 57 were excluded: 32 declined to take part, 12 were pregnant or lactating and thus ineligible and 13 had incomplete anthropometric or questionnaire data. The final analysis sample comprised 563 individuals, corresponding to 84.0% of all eligible selected individuals and to 94.6% of those who were contacted. The participant selection process and distribution by place of residence are summarised in figure 2.

**Figure 2:**
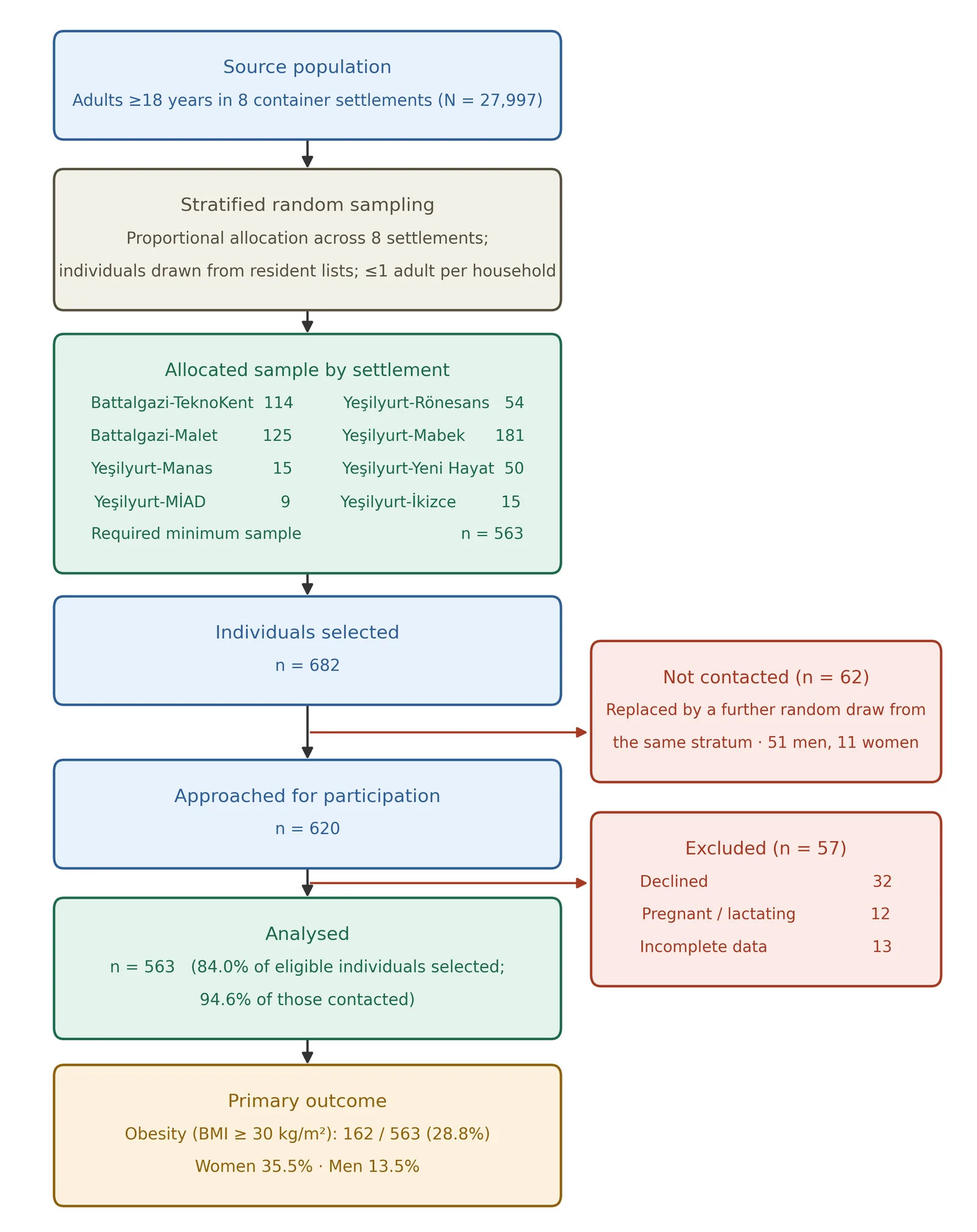
Flow diagram of participant selection. The source population comprised 27 997 adults aged ≥18 years permanently residing in eight container settlements established in Malatya after the 6 February 2023 Kahramanmaraş earthquakes. The required minimum sample of 563 was distributed across settlements by proportional stratified random sampling (Battalgazi-TeknoKent, 114; Battalgazi-Malet, 125; Yeşilyurt-Manas, 15; Yeşilyurt-MİAD, 9; Yeşilyurt-Rönesans, 54; Yeşilyurt-Mabek, 181; Yeşilyurt-Yeni Hayat, 50; Yeşilyurt-İkizce, 15), with named individuals drawn from resident lists and no more than one adult retained per household. Of the 682 individuals selected, 62 could not be contacted during daytime fieldwork and were replaced by a further random draw from the same stratum. A total of 620 adults were approached during fieldwork between June and October 2025; 57 were excluded (declined to participate, n=32; pregnant or lactating, n=12; incomplete anthropometric or questionnaire data, n=13). The final analytic sample comprised 563 individuals (84.0% of the eligible individuals selected and 94.6% of those contacted), of whom 162 met the criterion for obesity (body mass index ≥30 kg/m²), corresponding to an overall prevalence of 28.8% (35.5% in women and 13.5% in men). BMI, body mass index.

### Data collection

Data were collected through face-to-face interviews conducted by trained research staff in private settings free from distractions. The questionnaire covered the following topics: (i) sociodemographic characteristics, (ii) self-reported history of chronic illness and earthquake-related physical injury, (iii) lifestyle and behavioural variables (smoking, alcohol consumption, physical activity, eating speed and recalled childhood weight) and (iv) the usual frequency of consumption of seven food groups (red meat, white meat, vegetables, baked goods/starch-based foods, dairy products, packaged or ready-made foods and sugary drinks) (in ordinal categories). The dietary questionnaire was not designed to estimate quantitative energy or macronutrient intake. Childhood overweight was assessed using a single self-reported item; recall bias was acknowledged in the limitations section. The questionnaire was compiled by the research team from items used in national health surveys and from the post-disaster obesity literature and was reviewed before fieldwork by five specialists, four in public health and one in nutrition and dietetics, whose comments were incorporated. It was then piloted with 30 medical interns to check wording, response categories, item order and administration time; minor changes of wording followed, and the mean administration time was approximately 15 min. The pilot served feasibility and face validity only: it did not involve residents of the container settlements, and no formal psychometric validation of the instrument was undertaken. The complete questionnaire, including the exact wording of the eating-speed and childhood-weight items, is provided as online supplemental file 1.

### Anthropometric measurements

Height and weight measurements were taken by trained research staff, following standardised procedures, using a pre-calibrated digital scale with an integrated stadiometer. Height was recorded to the nearest millimetre while the participant stood upright, barefoot, with their head aligned with the Frankfurt horizontal plane; weight was recorded to the nearest 0.1 kg while the participant was wearing light clothing and was barefoot. BMI was calculated by dividing weight (kg) by the square of height (m²).

The WHO’s BMI cut-off points (kg/m²) for adults were applied^1^: <18.5, underweight; 18.5–24.9, normal weight; 25.0–29.9, overweight; and ≥30.0, obese. The primary outcome of the regression analysis was obesity status (≥30 kg/m²).

### Patient and public involvement

Patients and members of the public were not formally involved in the study design or interpretation. The research question arose from clinical concerns identified during the provision of primary healthcare services in residential settings. A simplified Turkish summary of the main findings will be shared with local authorities and the Malatya Provincial Health Directorate for distribution to residents via existing primary healthcare and community channels.

### Statistical analysis

Statistical analyses were performed using IBM SPSS Statistics V.22.0 (IBM Corp., Armonk, NY, USA). As continuous variables did not meet the normality assumption (Shapiro–Wilk test, p<0.05), they were expressed as median and IQR. Categorical variables were presented as frequency and percentage. The Mann–Whitney U test was used for comparisons between two groups, and the χ2 test was applied to assess associations between categorical variables. Post-hoc analysis was conducted for multiple group comparisons. Binary logistic regression analysis (enter method) was performed to identify factors associated with obesity; crude and adjusted ORs with 95% CIs are reported. Model fit was assessed using the Hosmer–Lemeshow test, and discriminatory power was evaluated by receiver operating characteristic (ROC) curve analysis. All covariates were entered simultaneously rather than selected into a prediction model, and the model was neither internally nor externally validated; the area under the curve therefore describes apparent, in-sample discrimination, and no diagnostic cut-off is proposed. Statistical significance was set at p<0.05. Figures were generated using Python programming language with matplotlib and seaborn libraries. Analyses were restricted to participants with complete data on all variables of interest (complete-case analysis). Prevalence estimates are reported with 95% CIs calculated by the Wilson score method, and the difference in prevalence across settlements was tested with the χ2 test. Because a large number of bivariate tests was carried out, the p values underlying table 1 were additionally corrected with the Benjamini–Hochberg procedure at a false discovery rate of 5% (online supplemental table S2). The variables entered into the multivariable model were specified before analysis and comprised sociodemographic characteristics (age group, sex, education, employment, household income and having children), clinical variables (chronic disease and recalled childhood weight status) and behavioural variables (dietary adequacy, number of daily meals, eating speed, vegetable consumption and smoking); age was entered in three categories to allow for a non-linear relationship with body weight and keep the model interpretable in a primary care setting. Multicollinearity was assessed with variance inflation factors, all of which were <2.6. Three sensitivity analyses were then performed: settlement was added to the model as a fixed effect, standard errors were re-estimated treating the eight settlements as clusters and prevalence ratios were estimated by modified Poisson regression with a robust variance estimator; because only eight clusters were available and conventional sandwich estimators are unreliable with so few clusters, the cluster-robust analysis is reported as exploratory, and greater weight is given to the settlement fixed-effect model because ORs depart from prevalence ratios when the outcome is common (online supplemental table S3). Prevalence was also standardised directly to an equal sex distribution, which approximates that of the Turkish adult population, with CIs obtained from 5000 bootstrap resamples.

**Table 1:** χ2 analysis of factors associated with obesity

		Obese (n=162)		P*
		N	%	
Age group, years	18–34	21	13.2†	**<0.001**
	35–54	79	31.6†	
	55–100	62	40.3†	
Sex	Female	139	35.5	**<0.001**
	Male	23	13.5	
Marital status	Married	127	30.6	0.109
	Single/other	35	23.6	
Education	Below high school	126	36.5	**<0.001**
	High school and above	36	16.5	
Employment	Employed	27	16.1	**<0.001**
	Unemployed	135	34.2	
Income (TRY)	0–22 104	125	36.0	**<0.001**
	>22 105	37	17.1	
Social security	Yes	106	26.4	0.054
	No	56	34.6	
Children	Yes	140	31.4	**0.007**
	No	22	18.8	
Childhood weight status	Underweight or normal	110	25.7	**0.004**
	Overweight/obese	52	38.5	
Presence of chronic disease	Yes	102	41.3	**<0.001**
	No	60	19.0	
Physical injury during earthquake	Yes	13	40.6	0.127
	No	149	28.1	
Adequate and balanced nutrition	Yes	75	24.3	**0.009**
	No	87	34.3	
Number of daily meals	≤2 meals/day	109	32.3	**0.022**
	≥3 meals/day	53	23.5	
Regular breakfast consumption	Yes	118	29.8	0.409
	No	44	26.3	
Eating speed	Fast	49	37.4	**0.013**
	Normal or slow	113	26.2	
Red meat consumption (servings/week)	>3	13	23.2	0.333
	≤3	149	29.4	
White meat consumption (servings/week)	>3	13	26.0	0.650
	≤3	149	29.0	
Vegetable consumption (servings/week)	>3	85	25.0	**0.015**
	≤3	77	34.5	
Pastry/dough consumption (servings/week)	>3	27	24.3	0.248
	≤3	135	29.9	
Dairy consumption (servings/week)	>3	114	27.9	0.441
	≤3	48	31.2	
Packaged food consumption (servings/week)	>3	11	19.3	0.096
	≤3	151	29.8	
Sugary beverage consumption (servings/week)	>3	21	24.4	0.332
	≤3	141	29.6	
Regular exercise	Yes	25	27.5	0.764
	No	137	29.0	
Physical activity area availability	Yes	62	30.0	0.638
	No	100	28.1	
Change in physical activity habits	Decreased	59	30.3	0.839
	Increased	19	27.1	
	Unchanged	84	28.2	
Smoking status	Yes	45	22.1	**0.008**
	No	117	32.6	
Alcohol use	Yes	3	13.0	0.089
	No	159	29.4	
Change in dietary habits in container city	Became healthier	4	33.3	0.531
	Remained the same	73	31.1	
	Became unhealthier	85	26.9	
Effect of container city living on weight control	Caused weight gain	90	41.9	**<0.001**
	Caused weight loss	21	15.7	
	No effect	51	23.8	

* χ2test was applied.

† Different superscript letters indicate statistically significant differences between age groups (p<0.05).

‡ Bold values indicate statistical significance (p<0.05).

### Bias minimisation

Efforts to minimise bias included the use of pre-calibrated digital equipment for anthropometric measurement, trained research staff for interviewing and measurement, private and distraction-free interview settings to protect confidentiality and reduce social-desirability bias and direct measurement of height and weight rather than self-report. Because interviews were conducted during the day, non-contact was more frequent among men; individuals who could not be reached were replaced by a further random draw rather than by whoever happened to be available at the address, and the resulting imbalance is addressed through sex-specific and sex-standardised estimates.

### Ethics

The study protocol was approved by the Non-Interventional Clinical Research Ethics Committee of the Faculty of Health Sciences, İnönü University (decision date: 3 December 2024; session 20; decision no. 2024/6812). All procedures complied with the Declaration of Helsinki (2013 revision).^24^ Before each interview, the study aim, voluntary nature, right to withdraw and anonymous handling of data were explained by trained research staff. Informed verbal consent was then obtained from each participant. Verbal rather than written consent was explicitly approved by the ethics committee given the post-disaster setting: many participants had lost identifying documents, signed paperwork was an avoidable burden in a displaced population and absence of a signed link offered additional protection against identification. Consent and its date were recorded by the interviewer; no personal identifiers were collected at any stage, and all data were processed anonymously.

## Results

Of the participants, 69.6% were female, 73.7% were married and 61.3% had an educational level below high school. Regarding earthquake damage, 71.9% of participants had lived in severely damaged or completely collapsed homes. The median duration of container city residence was 25.0 months (IQR 24.0–28.0), and 85.6% of participants were permanent residents of the container city (table 2).

**Table 2:** Distribution of sociodemographic and earthquake-related characteristics of the participants

		N	%
Age, median (IQR)		43.0 (32.0–56.0)	
Age group	18–34	159	28.2
	35–54	250	44.4
	55–100	154	27.4
Sex	Female	392	69.6
	Male	171	30.4
Marital status	Married	415	73.7
	Single/other	148	26.3
Education	Below high school	345	61.3
	High school and above	218	38.7
Employment	Employed	168	29.8
	Unemployed	395	70.2
Income (TRY)	0–22 104	347	61.6
	>22 105	216	38.4
Social security	Yes	401	71.2
	No	162	28.8
Children	Yes	446	79.2
	No	117	20.8
Earthquake damage	None/minor	69	12.3
	Moderate	89	15.8
	Severe	237	42.1
	Collapsed	168	29.8
Physical injury	Yes	32	5.7
	No	531	94.3
Container residency frequency	Permanent	482	85.6
	Mostly here	60	10.7
	Rarely here	21	3.7
Container stay duration, median (IQR), months		25.0 (24.0–28.0)	
Household size, median (IQR)		4.0 (3.0–5.0)	

The median BMI of the participants was 26.8 (IQR 24.0–30.9) kg/m², with 27.8 (IQR 24.1–32.4) in females and 26.0 (IQR 23.9–28.3) in males (figure 3). Overall, 28.8% of the participants (95% CI 25.2 to 32.6) were classified as obese (≥30 kg/m²), with 35.5% (30.9–40.3) in females, 13.5% (9.1–19.4) in males, and 24.4% (21.1–27.9) after direct standardisation to an equal sex distribution. Among individuals who were obese, 57.4% met the criteria for class I, 32.7% for class II and 9.9% for class III obesity (table 3).

**Figure 3:**
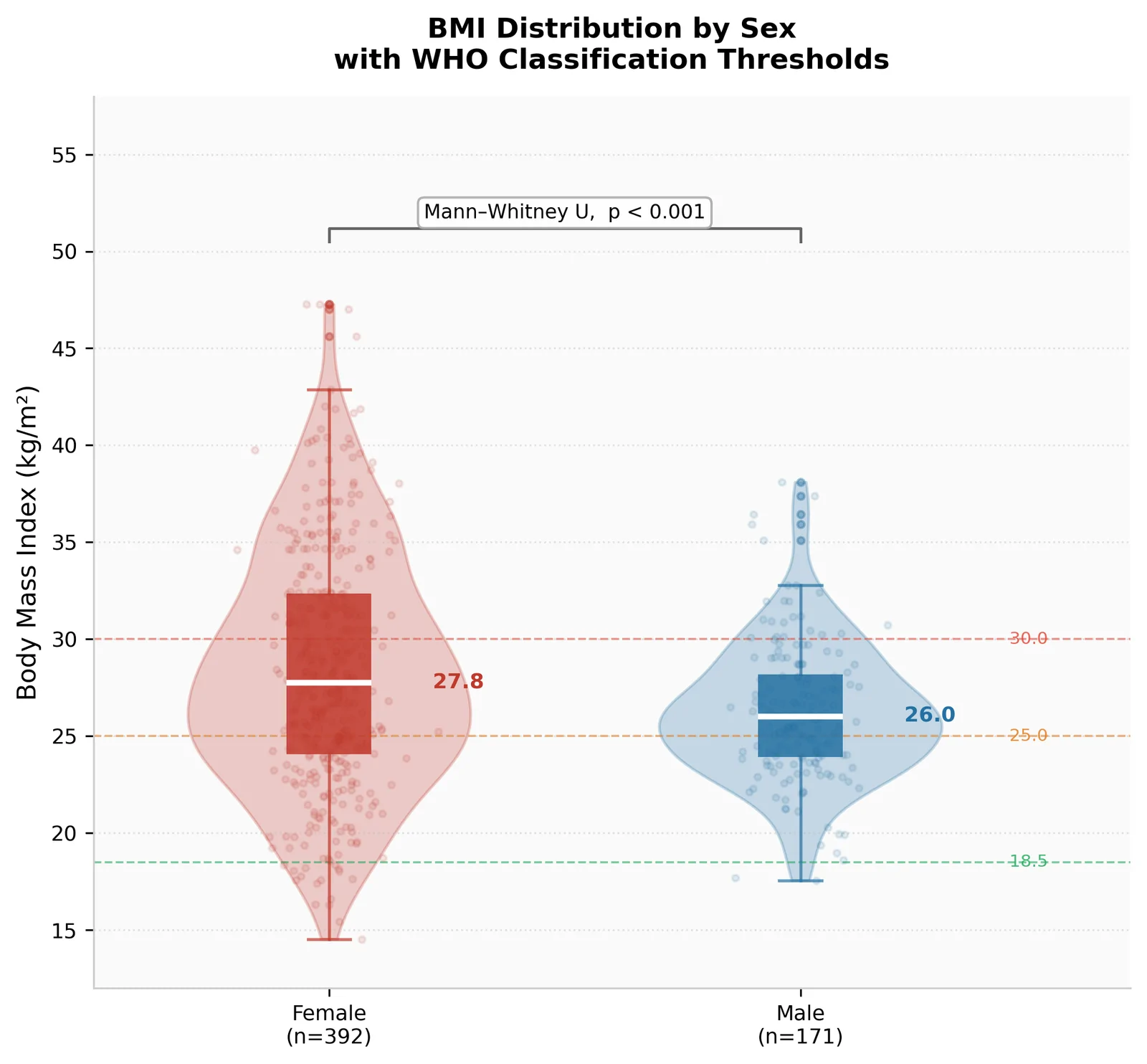
Distribution of body mass index (BMI) by sex with WHO classification thresholds. Combined violin and box plots show the distribution of BMI (kg/m²) among 392 female (red) and 171 male (blue) participants; individual observations are overlaid as jittered points. The white horizontal line within each box indicates the median (women 27.8 kg/m², men 26.0 kg/m²), and box edges represent the IQR. Horizontal dashed reference lines indicate the WHO adult BMI cut-points: 18.5 (lower limit of normal weight), 25.0 (lower limit of overweight) and 30.0 (lower limit of obesity). The distribution is markedly shifted upward in women compared with men (Mann–Whitney U test, p<0.001).

**Table 3:** Distribution of body mass index and prevalence of obesity among participants

		Median (IQR)	
BMI		26.8 (24.0–30.9)	
Female		27.8 (24.1–32.4)	
Male		26.0 (23.9–28.3)	
Height (m)		1.6 (1.6–1.7)	
Weight (kg)		73.0 (64.0–83.0)	
		**n**	**%**
BMI category	Underweight (<18.5)	18	3.2
	Normal weight (18.5–24.9)	175	31.1
	Overweight (25.0–29.9)	208	36.9
	Obese (≥30.0)	162	28.8
Obesity degree	Class I (30.0–34.9)	93	57.4
	Class II (35.0–39.9)	53	32.7
	Class III (≥40.0)	16	9.9

Obesity degree percentages are calculated based only on participants who were obese (n=162).

Obesity prevalence varied across container settlements, from 18.8% (95% CI 13.8 to 25.1) in Mabek to 66.7% (41.7–84.8) in Manas; the difference across the eight sites was statistically significant (χ²=29.21, df=7, p<0.001) (figure 4). The intervals for Manas (n=15), İkizce (n=15) and MİAD (n=9) are wide, and site-specific estimates resting on fewer than 20 participants should be read as indicative only (online supplemental table S4).

**Figure 4:**
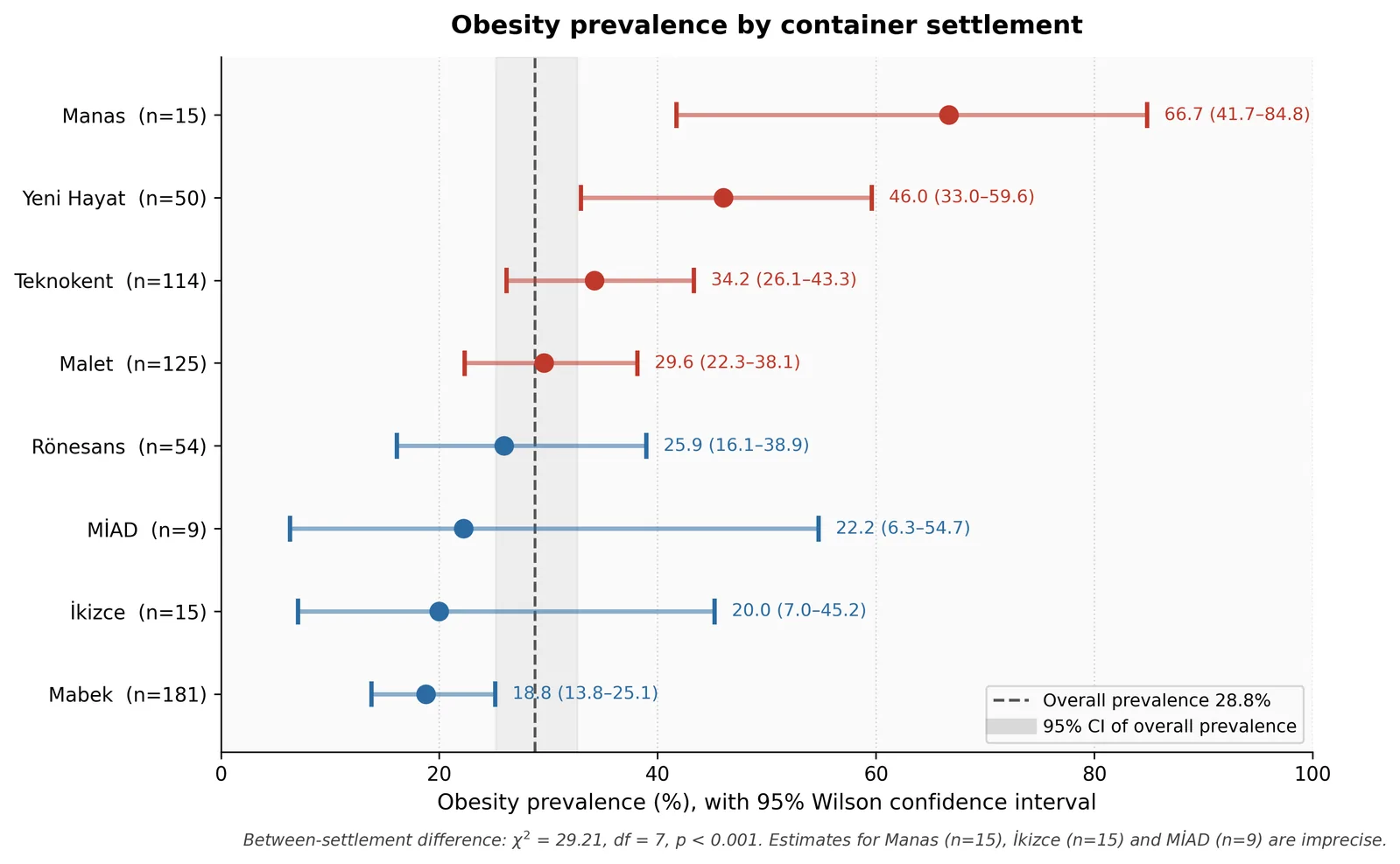
Obesity prevalence by container settlement. Point estimates with 95% Wilson CIs for the proportion of adults with body mass index ≥30.0 kg/m² in each of the eight container settlements, ordered from the lowest to the highest prevalence. The vertical dashed line marks the overall sample prevalence (28.8%) and the shaded band its 95% CI. Settlements with a prevalence below the overall value are shown in blue and those above it in red. The difference across settlements was statistically significant (χ²=29.21, df=7, p<0.001), but the estimates for Manas (n=15), İkizce (n=15) and MİAD (n=9) are imprecise. Sample sizes per settlement are given in parentheses.

Of the participants, 45.1% reported inadequate and unbalanced nutrition, and 59.9% consumed two or fewer meals per day. Among participants, 56.1% reported that their dietary habits had become unhealthier since living in the container city, and 38.2% indicated that container city living had caused them to gain weight. Regarding physical activity, 83.8% of participants did not exercise regularly, and 34.6% reported a decrease in their physical activity habits (table 4). Dietary consumption patterns across BMI categories are illustrated in figure 5.

**Table 4:** Dietary habits, physical activity status and container city living–related findings of the participants

		N	%
Adequate and balanced nutrition	Yes	309	54.9
	No	254	45.1
Number of meals per day	≤2	337	59.9
	≥3	226	40.1
Meal most frequently skipped	Breakfast	152	27.0
	Lunch	261	46.4
	Dinner	51	9.1
	No meal skipped	99	17.6
Regular breakfast consumption	Yes	396	70.3
	No	167	29.7
Eating speed	Fast	131	23.3
	Normal or slow	432	76.7
Frequent eating outside home	Yes	86	15.3
	No	477	84.7
Primary cooking method	Frying	123	21.8
	Sautéing	104	18.5
	Boiling	152	27.0
	Baking/oven	184	32.7
Red meat consumption (servings/week)	>3	56	9.9
	≤3	507	90.1
White meat consumption (servings/week)	>3	50	8.9
	≤3	513	91.1
Vegetable consumption (servings/week)	>3	340	60.4
	≤3	223	39.6
Pastry/dough consumption (servings/week)	>3	111	19.7
	≤3	452	80.3
Dairy consumption (servings/week)	>3	409	72.6
	≤3	154	27.4
Packaged food consumption (servings/week)	>3	57	10.1
	≤3	506	89.9
Sugary beverage consumption (servings/week)	>3	86	15.3
	≤3	477	84.7
Change in dietary habits in container city	Became healthier	12	2.1
	Remained the same	235	41.7
	Became unhealthier	316	56.1
Effect of container city living on weight control	Caused weight gain	215	38.2
	Caused weight loss	134	23.8
	No effect	214	38.0
Regular exercise	Yes	91	16.2
	No	472	83.8
Physical activity area availability	Yes	207	36.8
	No	356	63.2
Change in physical activity habits	Decreased	195	34.6
	Increased	70	12.4
	Unchanged	298	52.9
Smoking status	Yes	204	36.2
	No	359	63.8
Alcohol use	Yes	23	4.1
	No	540	95.9

**Figure 5:**
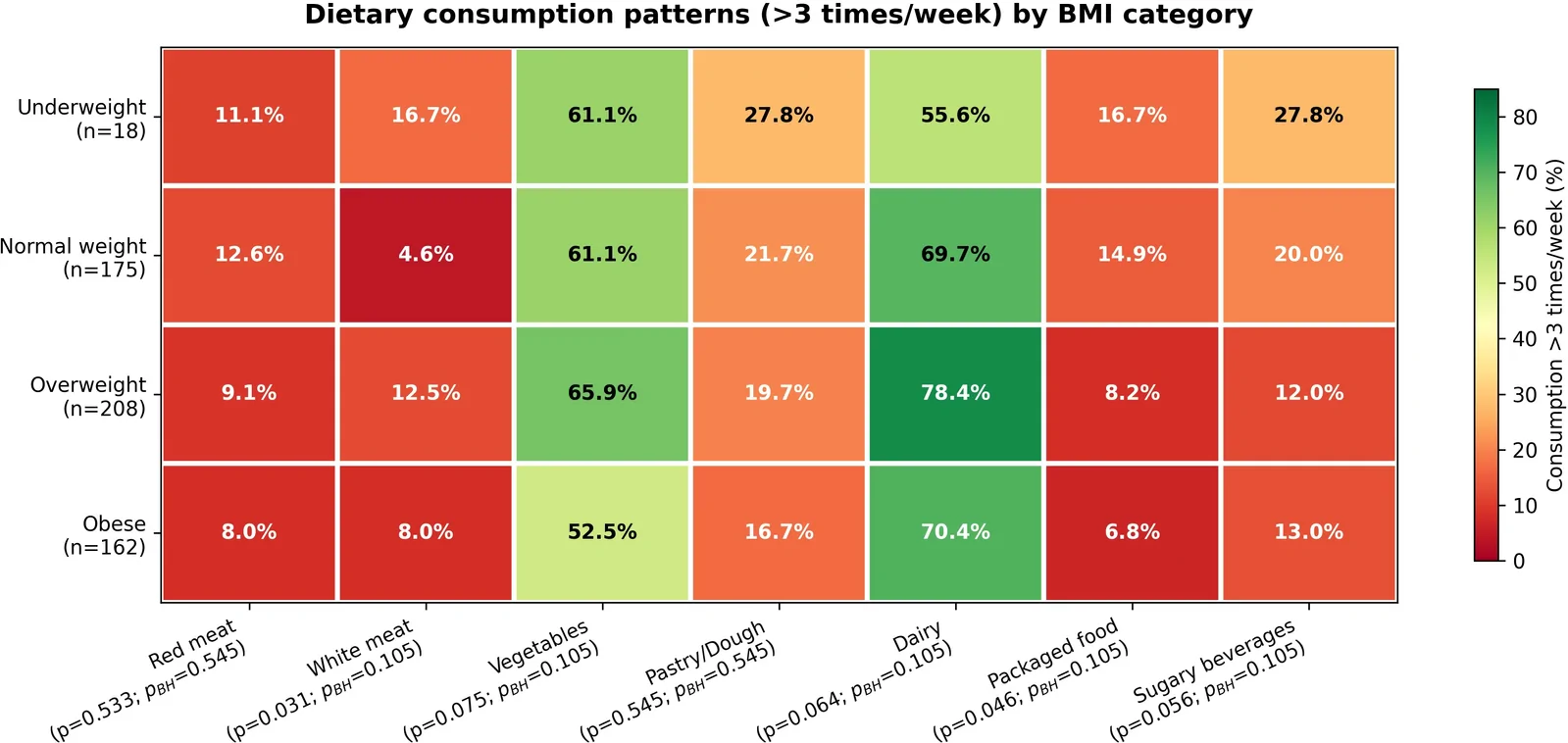
Dietary consumption patterns (>3 times/week) by body mass index (BMI) category. Heatmap showing the proportion of participants reporting consumption more than three times per week of each of seven food groups (red meat, white meat, vegetables, pastry/dough-based foods, dairy products, packaged or ready-to-eat foods and sugar-sweetened beverages), stratified by BMI category (underweight, normal weight, overweight and obese). Cell values represent the percentage within each BMI category. Colour intensity reflects the consumption frequency, ranging from low (red) to high (green). p values from Pearson’s χ2 test are reported in the column labels together with p values corrected for multiple testing by the Benjamini–Hochberg procedure; no dietary item remained statistically significant after correction. The underweight group (n=18) should be interpreted with caution owing to the small sample size.

Age group, sex, educational level, employment status, income level, having children, childhood weight status, presence of chronic disease, adequate nutrition, number of daily meals, eating speed, vegetable consumption and smoking status were statistically significantly associated with obesity (p<0.05). The prevalence of obesity was 35.5% in females, 13.5% in males and 40.3% in the ≥55 age group compared with 13.2% in the 18–34 age group. Post-hoc analysis revealed that the ≥55 age group differed significantly from both the 18–34 and 35–54 age groups (table 1). After Benjamini–Hochberg correction across the 29 tests, 13 of the 14 associations remained significant; only the number of daily meals moved above the threshold (online supplemental table S2).

Binary logistic regression analysis was performed to identify factors independently associated with obesity. The overall model was statistically significant (p<0.001), with a Nagelkerke R² of 0.267. The Hosmer–Lemeshow test confirmed adequate model fit (χ²=10.544, df=8, p=0.229). ROC curve analysis indicated acceptable apparent in-sample discrimination (AUC=0.775, 95% CI 0.732 to 0.818, p<0.001); the model was not validated, and no cut-off is proposed (figure 6).

**Figure 6:**
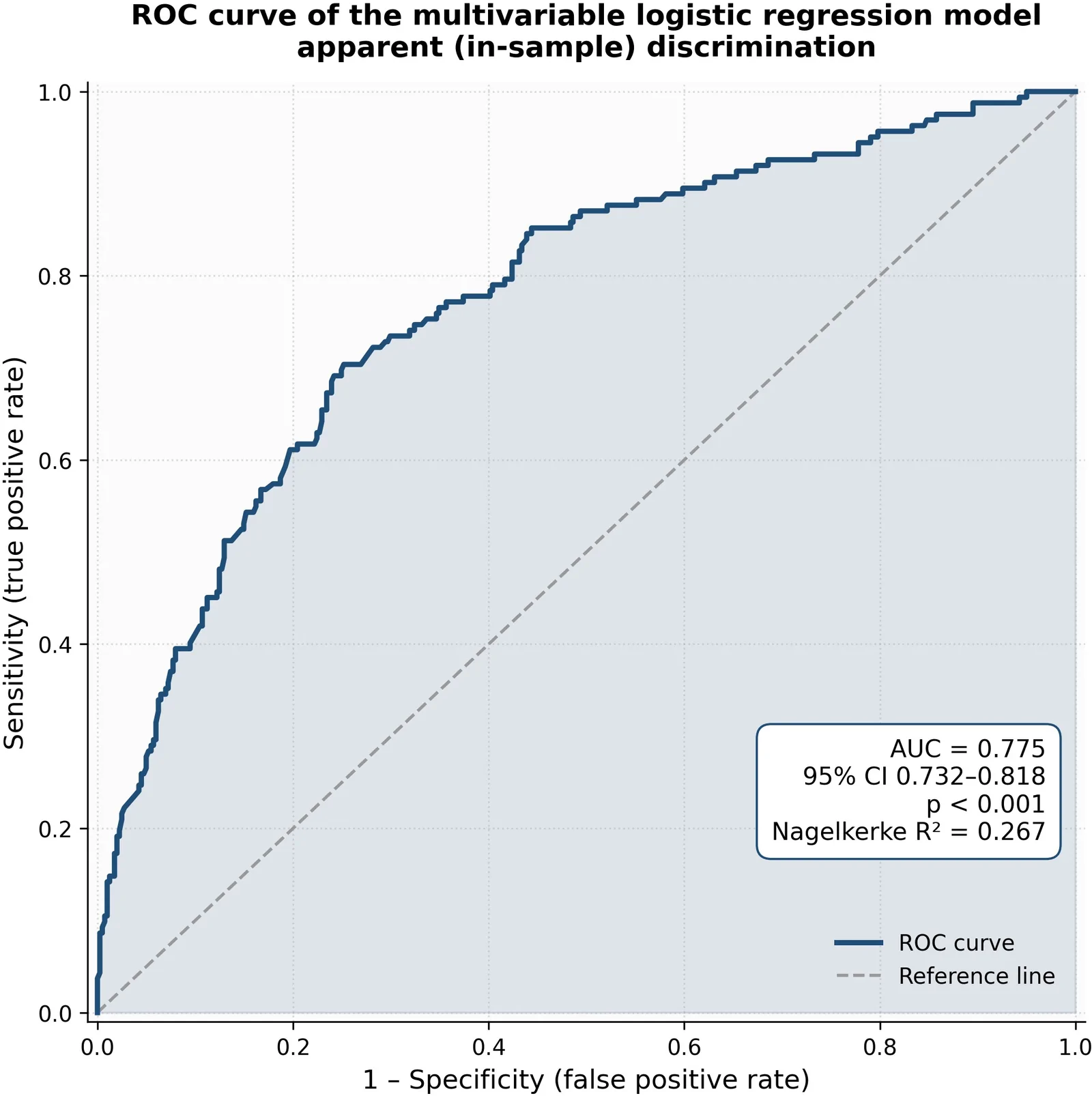
Receiver operating characteristic (ROC) curve of the multivariable logistic regression model. The solid line represents the ROC curve of the model, and the diagonal dashed line corresponds to chance discrimination (AUC=0.50). AUC=0.775 (95% CI 0.732 to 0.818, p<0.001); Nagelkerke R²=0.267. All covariates were entered simultaneously rather than selected into a prediction model, and no internal or external validation was performed; the curve therefore describes apparent in-sample discrimination, and no diagnostic cut-off is proposed. Six variables were independently associated with obesity in the model: female sex, age ≥55 years, fast eating, self-reported childhood overweight, presence of chronic disease and low household income. AUC, area under the curve.

Regarding adjusted ORs, female sex showed the strongest independent association with obesity (AOR=3.458, 95% CI 1.857 to 6.437, p<0.001). Compared with the 18–34 age group, both the 35–54 age group (AOR=2.141, 95% CI 1.138 to 4.029, p=0.018) and ≥55 age group (AOR=2.436, 95% CI 1.166 to 5.090, p=0.018) had significantly higher odds of obesity. Fast eating speed was associated with 2.3-fold higher odds of obesity (AOR=2.276, 95% CI 1.386 to 3.736, p=0.001). The presence of chronic disease (AOR=1.853, 95% CI 1.166 to 2.944, p=0.009), childhood overweight history (AOR=1.927, 95% CI 1.208 to 3.071, p=0.006) and low income level (AOR=1.730, 95% CI 1.047 to 2.858, p=0.032) were also independently associated with obesity. Educational level, employment status, having children, dietary adequacy, number of daily meals, vegetable consumption and smoking status lost statistical significance in the adjusted analysis (table 5).

**Table 5:** Logistic regression analysis of factors associated with obesity

Variable	Crude			Adjusted		
	OR	95% CI	P	OR	95% CI	P
Age group						
18–34 (ref)	1	–	–	1	–	–
35–54	3.036	1.785 to 5.162	<0.001	2.141	1.138 to 4.029	0.018
55–100	4.429	2.528 to 7.759	<0.001	2.436	1.166 to 5.090	0.018
Sex						
Male (ref)	1	–	–	1	–	–
Female	3.535	2.175 to 5.745	<0.001	3.458	1.857 to 6.437	<0.001
Educational level						
High school and above (ref)	1	–	–	1	–	–
Below high school	2.909	1.912 to 4.424	<0.001	1.384	0.792 to 2.417	0.253
Employment status						
Employed (ref)	1	–	–	1	–	–
Unemployed	2.712	1.710 to 4.301	<0.001	1.058	0.568 to 1.969	0.860
Monthly income (TRY)						
>22 105 (ref)	1	–	–	1	–	–
0–22 104	2.724	1.796 to 4.131	<0.001	1.730	1.047 to 2.858	0.032
Having children						
No (ref)	1	–	–	1	–	–
Yes	1.976	1.192 to 3.274	0.008	1.143	0.599 to 2.181	0.685
Childhood weight status						
Underweight/normal (ref)	1	–	–	1	–	–
Overweight/obese	1.811	1.203 to 2.726	0.004	1.927	1.208 to 3.071	0.006
Chronic disease						
Absent (ref)	1	–	–	1	–	–
Present	3.001	2.056 to 4.382	<0.001	1.853	1.166 to 2.944	0.009
Adequate and balanced nutrition						
Yes (ref)	1	–	–	1	–	–
No	1.625	1.126 to 2.346	0.010	1.149	0.751 to 1.758	0.521
Number of daily meals						
≥3 meals (ref)	1	–	–	1	–	–
≤2 meals	1.560	1.064 to 2.289	0.023	1.502	0.966 to 2.337	0.071
Eating speed						
Normal/slow (ref)	1	–	–	1	–	–
Fast	1.687	1.115 to 2.552	0.013	2.276	1.386 to 3.736	0.001
Vegetable consumption						
>3 times/week (ref)	1	–	–	1	–	–
≤3 times/week	1.582	1.094 to 2.289	0.015	1.475	0.967 to 2.251	0.071
Smoking status						
No (ref)	1	–	–	1	–	–
Yes	1.708	1.148 to 2.543	0.008	1.313	0.818 to 2.110	0.260

Nagelkerke R²=0.267; Hosmer–Lemeshow test: χ²=10.544, df=8, p=0.229; apparent AUC=0.775 (95% CI 0.732 to 0.818), p<0.001

The pattern of associations was stable across the sensitivity analyses (online supplemental table S3). With settlement entered as a fixed effect, the estimates for sex, age, eating speed and childhood weight status were essentially unchanged, the association with chronic disease was attenuated (AOR 1.63, 95% CI 1.01 to 2.63), and the association with low household income was no longer statistically significant (AOR 1.48, 95% CI 0.88 to 2.47). Cluster-robust standard errors, treating the eight settlements as clusters, left all six associations significant, although with only eight clusters this analysis is exploratory and the settlement fixed-effect model carries more weight. Modified Poisson regression yielded prevalence ratios closer to unity than the corresponding ORs, as expected for a common outcome: female sex 2.26 (95% CI 1.39 to 3.67), age ≥55 years 1.83 (95% CI 1.07 to 3.14), fast eating 1.52 (95% CI 1.21 to 1.92), childhood overweight 1.47 (95% CI 1.15 to 1.87), chronic disease 1.45 (95% CI 1.07 to 1.95) and low household income 1.44 (95% CI 1.03 to 2.00). None of the settlement-related conditions reported by participants—adequacy of food storage, access to a market, availability of an area for physical activity or household size—was independently associated with obesity (online supplemental table S5).

## Discussion

### Principal findings

In this population-based cross-sectional study conducted on 563 adults living in eight container settlements established in Malatya following the 6 February 2023 Kahramanmaraş earthquakes, the prevalence of obesity was found to be 28.8% and the median BMI was 26.8 kg/m² (IQR 24.0–30.9). The burden of obesity was concentrated among women (35.5% compared with 13.5% among men) and varied between settlements (from 18.8% in Mabek to 66.7% in Manas). After adjustment, six factors were independently associated with obesity: female sex, age ≥55 years, eating quickly, being overweight in childhood (self-reported), chronic illness and low household income (table 5). Unlike the other five, the association with low household income did not persist once settlement was included in the model. The model showed acceptable apparent discrimination (AUC 0.775, 95% CI 0.732 to 0.818), which reflects fit within this sample rather than validated predictive performance. To our knowledge, this is the first population-based study to assess obesity in adults living in earthquake-related container settlements in the Eastern Mediterranean region and examine the under-researched 24–32-month subacute-to-chronic phase of displacement.

### Comparison with other studies

The prevalence rate of 28.8% is lower than the national figure of 32.1%^5^ and is largely consistent with the TURDEP-II estimate of 31.5%,^7^ but the comparison requires care. Women made up 69.6% of our sample against an almost equal sex distribution nationally, and because obesity was far more common among women than among men (35.5% vs 13.5%), the crude figure is displaced upwards by the composition of the sample rather than downwards. Standardised to an equal sex distribution, prevalence falls to 24.4% (95% CI 21.1 to 27.9), below the national estimates, so comparisons with national data should rest on the standardised value and sex-specific figures (online supplemental table S6). The 66.7% recorded in Manas exceeds provincial figures reported for the country, although it rests on 15 participants and its CI runs from 41.7% to 84.8%. A systematic review of 17 cohorts (229 058 participants) concluded that natural disasters consistently increase obesity, with displacement, restricted access to fresh food, a sedentary lifestyle and psychosocial stress being the primary mediating factors.^14^ In cohorts following the 2011 Great East Japan Earthquake, an increase from 25.0% to 35.1% was reported among displaced survivors within approximately 30 months, whereas this increase remained stable in the non-displaced control group,^10^ a trend that persisted in 5-year and 7-year follow-ups.^12 13^ The level measured herein was close to the post-displacement endpoint of the Hikichi cohort; without a pre-disaster baseline, however, this similarity can only be read as consistent with the pattern described in the East Asian cohorts and not as evidence of it in our setting.^15^

The most pronounced finding is the magnitude of the sex disparity: women retained almost 3.5 times the odds of obesity after adjustment. The Turkish Health Survey 2022 reports a national gap of 23.6% versus 16.8%,^6^ and modelling to 2050 anticipates 39.7% in women versus 22.0% in men.^25^ Across the Middle East and North Africa, the female–male gap averages 10.3 percentage points and tracks female labour-market participation rather than biology alone.^26^ The 22-percentage-point gap measured here far exceeds both averages, compatible with three structural exposures that may fall harder on women, none of which was measured here and each of which remains a hypothesis for further work: a confined 21 m² unit concentrating food preparation and domestic labour on female members; an unemployed proportion of 70.2% patterned by sex^27^; and a participant-reported shift towards energy-dense, shelf-stable items rather than fresh produce. Part of the crude gap may also reflect who could be interviewed during the day, although the adjusted estimate does not depend on the sex composition of the sample. Where the food environment is reduced to what fits in a 21 m² unit, the sex-responsive approach to food environments set out by Adams *et al*^9^ is directly relevant.

The age trend was steep; among adults aged ≥55 years, the adjusted odds were more than double those of people aged 18–34. This pattern is consistent with the well-defined changes in body composition that occur with age^4^ and is likely to be further exacerbated in settings where older residents have reduced access to outdoor walking areas and a heavier burden of chronic disease. Chronic disease was independently associated with obesity, although the cross-sectional design precludes determining causality. The association with low income is consistent with the long-standing pattern in Turkey of obesity following socio-economic disadvantage,^27^ although it was the least robust of the six findings and did not survive adjustment for settlement; in the post-disaster context, this pathway is further reinforced by International Labour Organization and International Organization for Migration evidence showing that household income, employment and food security in affected provinces remained well below pre-disaster levels until the second year of recovery^21^ and is narrowing the range of food choices towards cheaper, energy-dense foods.^8^

Eating quickly showed one of the strongest behavioural associations, with more than a twofold increase in the adjusted odds. This falls within the range reported in a meta-analysis of 23 studies (pooled OR 2.15, 95% CI 1.84 to 2.51)^28^ and a more recent systematic review with similar findings.^29^ Meal duration may plausibly be shorter in a container setting because of overcrowding and the absence of a dedicated dining area, although neither was measured in this study and this remains a hypothesis to be tested; eating speed is also one of several obesity-related behaviours that primary care services could address through brief counselling^30^ . Self-reported childhood overweight has remained associated with adult obesity; although a single recalled item cannot replace measured childhood anthropometry, the direction and magnitude are consistent with cohort evidence.^3^ Its persistence after full adjustment is compatible with an influence of early-life weight that operates independently of current circumstances, although our data cannot separate this from the conditions of container living.

The difference between the lowest and the highest settlement prevalence (18.8% in Mabek vs 66.7% in Manas) was statistically significant (p<0.001), but the extremes rest on small numbers, and their CIs are correspondingly wide. Adding settlement as a fixed effect left most associations unchanged while attenuating the association with household income, which suggests that part of the variation between sites reflects differences in the composition of their populations rather than the sites themselves. None of the settlement-related conditions recorded from participants was independently associated with obesity, and no objective measurement of the built or food environment was available; heterogeneity of this order has rarely been described in displaced populations,^15^ and it is better treated as a question for multilevel studies with environmental measurement at settlement level than as evidence of a settlement effect.

### Strengths and limitations

Strengths include the population-based sampling frame covering all eight Malatya container settlements, proportional allocation, participation by 84.0% of the eligible adults selected, measured anthropometry and fieldwork in the under-investigated 24–32-month window.^16^ The model achieved acceptable calibration and apparent discrimination, and the six independently associated variables are biologically plausible. Limitations follow from the design: the cross-sectional structure precludes causal inference, and no pre-disaster baseline is available, so estimates reflect prevalence under displacement rather than change attributable to it. We did not include a non-displaced comparison group, so the prevalence cannot be clearly attributed to container-city living as opposed to broader earthquake exposure or pre-existing regional patterns. Several behavioural exposures (childhood weight, eating speed and dietary patterns) were captured by single self-report items and are subject to recall and social-desirability bias. Dietary data were habitual frequency rather than quantitative intake. The study is geographically limited to one province.^22^ 62 selected individuals could not be contacted during daytime fieldwork, and 51 of them were men, so women are over-represented in the achieved sample; crude estimates are therefore accompanied by sex-specific and sex-standardised values, but residual non-contact bias cannot be ruled out. Settlement conditions were described by participants rather than measured objectively, and the three smallest settlements contributed very few participants for stable site-specific estimates. The questionnaire was piloted among medical interns rather than among adults resembling the study population, so comprehension by respondents with lower educational attainment was not formally assessed, although every item was administered by a trained interviewer who could clarify it. BMI does not capture body fat distribution; central adiposity was not measured.

### Implications and future research

These findings have three implications. First, obesity should be treated as a present problem of the recovery phase, not a future concern of permanent rehousing. The high mental-health burden in the same provinces—pooled post-traumatic stress disorder prevalence of 41%^31^ and persistently elevated symptoms at 12 months in Malatya^32^—should be addressed alongside, not separately from, weight and eating behaviour. Second, the population at greatest risk is identifiable in advance: women aged ≥55 years with chronic disease and low income had the highest prevalence and are a reasonable priority group, although the contribution of income specifically was not robust to adjustment for settlement and our data cannot indicate how much benefit an intervention would produce. On-site primary care is well placed to deliver brief behavioural counselling and triage women with multiple risk factors for more intensive support. Third, the structural determinants—unit size, cooking and storage infrastructure, outdoor activity space and distance to fresh-food markets—sit largely outside the health sector and plausibly constrain what behavioural intervention alone can achieve, though we did not measure them objectively and their contribution remains to be tested. Future research should pursue: longitudinal cohorts with baseline anthropometry, site-level mapping of food and activity environments paired with individual outcomes and pragmatic intervention trials embedded in on-site primary care evaluating sex-responsive support against usual care.

## Conclusion

Among adults living in eight Malatya container settlements 24–32 months after the 2023 Kahramanmaraş earthquakes, obesity prevalence was 28.8% (95% CI 25.2 to 32.6), or 24.4% after standardisation to an equal sex distribution, with marked concentration among women (35.5% vs 13.5%) and a wide spread between settlements (18.8%–66.7%). Six factors—female sex, age≥55, fast eating, childhood overweight, chronic disease and low income—were independently associated with obesity, although the income association was sensitive to adjustment for settlement; apparent model discrimination was acceptable (AUC 0.775). This profile supports targeted intervention through on-site primary care, prioritising older women with chronic disease and limited income; the variation between settlements should be examined directly, with environmental measurement at settlement level. Longitudinal cohorts with pre-disaster baselines and pragmatic intervention trials embedded in primary care are needed to translate these findings into an evidence base for comparable post-disaster contexts.

## Supplementary material

10.1136/bmjopen-2026-122076 online supplemental file 1

10.1136/bmjopen-2026-122076 online supplemental file 2

## Data Availability

Data are available upon reasonable request.
