# Supplementary material for: Obesity among adults living in post-earthquake container settlements: a cross-sectional study in Malatya, Türkiye
Source: BMJ Open. 2026 Sep 23;16(9):e122076. doi: 10.1136/bmjopen-2026-122076 (PMC13629913; doi:10.1136/bmjopen-2026-122076)
Supplement: Supplementary file 1 [file bmjopen-2026-122076.reviewer_comments.pdf]

PEER REVIEW HISTORY

BMJ Open publishes all reviews undertaken for accepted manuscripts. Reviewers are asked to complete a checklist review form and are provided with free text boxes to elaborate on their assessment. These free text comments are reproduced below.

ARTICLE DETAILS

Title (Provisional)

Obesity Among Adults Living in Post-Earthquake Container Settlements: A Cross-Sectional Study in Malatya, Türkiye

Authors

kurt, osman; Aslan, Aleyna; Avcu, Ahmet Burak; Baran, Ayşe; Ozer, Ali; Genç, Metin Fikret; Kurt, Harun

---

VERSION 1 - REVIEW

**Reviewer**	**1**
**Name**	**Sun, Mingyao**
**Affiliation**	**Fujian Provincial Hospital, Geriatric Medicine**
**Date**	**20-Jun-2026**
**COI**	**None**

This is a clearly written, timely, and methodologically sound cross-sectional study addressing an important and under-investigated public-health question: the prevalence and determinants of obesity among adults living in post-earthquake container settlements in Malatya, Türkiye. The topic is novel for the Eastern Mediterranean region, and the large population-based sample with directly measured anthropometry strengthens the credibility of the findings. Overall, the manuscript meets the scientific and ethical standards expected for publication in BMJ Open, though a few sections could be clarified or expanded before acceptance.

Comments：

1.The Introduction provides sufficient epidemiological background, but could more explicitly clarify how this study fills gaps in existing literature—especially compared with other post-disaster obesity data from Japan and MENA.Consider highlighting in the final paragraph why the 24-32-month “sub-acute to chronic” window is an important analytic stage versus earlier or later phases.

Methods clarity

2.The sampling description is detailed; please add a brief justification for choosing a design effect of 2 and clarify whether clustering by settlement was considered in the analysis (although simple random sampling within strata is stated).

3.“Fast eating” and “childhood overweight” were self-reported; please include exact wording of these items in supplementary material for transparency.Specify whether any adjustment for multiple comparisons was made for the numerous chi-square tests.

4.The logistic-regression modelling appears appropriate and well reported, but given the stratified design, indicate whether sampling weights were applied.

5.Minor grammatical and stylistic edits would further tighten the prose; overall English quality is fully acceptable.

6.Ensure consistent reporting of decimal separators (e.g., 30.0 kg/m² throughout).

Cross-check reference formatting to conform fully with BMJ Vancouver style; some DOIs lack spaces before numbers.

**Reviewer**	**2**
**Name**	**Meneguzzo, Paolo**
**Affiliation**	**Università degli Studi di Padova, Neuroscience**
**Date**	**28-Aug-2026**
**COI**	**None**

The manuscript addresses an important and relatively understudied post-disaster population and has notable strengths, particularly the use of measured height and weight and the attempt at population-based sampling. However, several methodological and interpretative issues require attention before the findings can be considered robust.

- Sampling was performed from household lists, with one adult included per household, but it is unclear how the adult within each selected household was chosen. This is important because adults from larger households may have different inclusion probabilities, and selecting the person available at the time of contact could introduce substantial bias. Please describe the within-household selection procedure and clarify whether sampling weights were required/applied. The marked predominance of women in the sample (69.6%) makes this particularly relevant.

- Statistical analysis should reflect the sampling structure. The study used proportional stratification across eight settlements, yet the regression appears to ignore settlement and the sampling design. This is especially problematic given the reported prevalence range from 18.8% to 66.7% across settlements. Please consider accounting for settlement in the analysis and explain whether weighting or design-adjusted variance estimates are required. At minimum, a sensitivity analysis including settlement as a fixed effect would be informative.

- Please explain how variables were selected for the adjusted model and whether this was prespecified. The current Methods only state that an Enter logistic regression was used. Selection based primarily on univariable statistical significance would not provide an adequate confounder-control strategy. The authors should also report assessment of multicollinearity and justify the categorisation of continuous variables such as age.

- Obesity prevalence is relatively high (28.8%), so odds ratios should not be interpreted as risk ratios. For a cross-sectional prevalence study, prevalence ratios estimated using modified Poisson or log-binomial regression may be more interpretable. If logistic regression is retained, the manuscript should consistently refer to prevalence odds/associations rather than "risk", "risk factors", "predictors", or "determinants".

- Uncertainty around prevalence estimates should be shown. Please provide 95% confidence intervals for the overall prevalence and key subgroup estimates, particularly settlement-specific prevalences. Some settlements have very small samples, making the raw range potentially unstable. The assertion that the between-settlement difference is unlikely to represent sampling variability is too strong without formal analysis.

- The manuscript argues that the 28.8% crude prevalence may underestimate the burden when standardised by sex, despite women constituting 69.6% of the sample and having substantially higher obesity prevalence than men. Given the reported sex-specific prevalences (35.5% vs 13.5%), standardisation toward an approximately balanced sex distribution would actually reduce, not increase, the crude estimate. Comparisons with national estimates should therefore be revised, ideally using age- and sex-standardised estimates.

- The absence of pre-earthquake anthropometry or a non-displaced comparison group means the study cannot determine whether container residence increased obesity. Likewise, environmental characteristics such as cooking/storage infrastructure or access to activity areas were not analysed as determinants. Statements that the "obesogenic exposure of the container city" acts on prior susceptibility, that structural characteristics explain settlement differences, or that particular groups will obtain the "largest absolute benefit" from intervention go beyond the data. The manuscript itself appropriately acknowledges the lack of a comparison group, but this caution is not consistently carried into the Discussion, Abstract, and Conclusion.

- The English is acceptable for publication, although several passages in the Discussion would benefit from more neutral epidemiological wording rather than stronger causal or rhetorical formulations.

VERSION 1 - AUTHOR RESPONSE

Reviewer 1

We are grateful for the Reviewer's positive assessment and for six specific suggestions, all of which we have acted on.

1. The Introduction could clarify more explicitly how this study fills gaps in existing literature, and why the 24–32-month window is an important analytic stage.

The final paragraph of the Introduction already identified the gap; we have kept its structure and sharpened the statement of what the 24–32-month window represents, namely the period in which displaced households move from short-term coping to a settled routine, which sits between the acute-phase studies and the five- and seven-year follow-ups of the Japanese cohorts. The comparison with the Japanese and MENA evidence is developed in the Discussion, where we have also made clear that our data cannot demonstrate the change over time that those cohorts measured directly.

2. Please add a brief justification for the design effect of 2, and clarify whether clustering by settlement was considered in the analysis.

Both points are now addressed in Methods. A design effect of 2 was adopted as a conservative allowance for residual similarity between residents of the same settlement, which is customary in surveys of displaced populations where a single national frame is unavailable. We also state that the eight settlements were treated as strata rather than as sampled clusters, because every settlement in the province was included rather than a sample of them. Clustering has nevertheless been examined empirically: the intraclass correlation for obesity between settlements was 0.063, and we now report analyses with settlement as a fixed effect and with cluster-robust standard errors (Supplementary Table S3). Neither materially altered the findings, with the single exception noted under Reviewer 2, point 2.

3. "Fast eating" and "childhood overweight" were self-reported; please include exact wording in supplementary material. Specify whether any adjustment for multiple comparisons was made.

The exact wording of both items appears in Supplementary File 1 (items 47 and 32). On multiple testing, the p values underlying Table 4 have been corrected with the Benjamini–Hochberg procedure at a false discovery rate of 5%; the procedure is described in Methods and the full set of corrected values is given in Supplementary Table S2. Thirteen of the fourteen associations remained significant; only the number of daily meals moved above the threshold, and the Results text now says so. The same correction has been applied to the dietary comparisons shown in Figure 5, where no item remained significant after correction; the figure and its legend have been updated accordingly and the asterisks removed.

4. Given the stratified design, indicate whether sampling weights were applied.

They were not required, and we now explain why. The sample was allocated in proportion to the adult population of each settlement, so the design is approximately self-weighting across strata. Selection was made at the level of the individual, so there was no within-household selection step that would call for a household weight (see Reviewer 2, point 1). As a check we nevertheless repeated the analysis weighting each participant by household size: the prevalence estimate moved from 28.8% to 27.8% and no association changed in direction or materially in magnitude (Supplementary Table S4).

5. Minor grammatical and stylistic edits would tighten the prose.

The manuscript has been read through and edited for concision, and several passages in the Discussion have been rewritten in more neutral epidemiological wording, as Reviewer 2 also suggested.

6. Ensure consistent reporting of decimal separators and cross-check reference formatting against BMJ Vancouver style.

Decimal points are now used consistently throughout the text, tables and figures, and BMI cut-offs are given uniformly to one decimal place. The reference list has been checked against BMJ Vancouver style, and the spacing of DOIs has been corrected.

Reviewer 2

We thank the Reviewer for a detailed methodological critique. One of the points identified a genuine error in our interpretation, which we have corrected, and the remainder have led to analyses that strengthen the paper.

1. It is unclear how the adult within each selected household was chosen; describe the within-household selection procedure and clarify whether sampling weights were required.

Our original wording was misleading and we apologise for it. There was no within-household selection stage. The sampling frame was a list of registered adult residents held by each settlement authority, and named individuals were drawn from that list with a random number generator before any field contact, so the person to be interviewed was identified in advance. The rule that no more than one adult per household would be retained was a constraint on the draw rather than a selection stage; with an overall sampling fraction of about 2%, two adults from the same household were seldom drawn and the constraint was rarely binding. Selection probabilities were therefore approximately equal across eligible adults and design weights were not required. The Methods have been rewritten to state this, and a household-size weighted sensitivity analysis is reported in Supplementary Table S4. The predominance of women is explained separately under point 6.

2. The regression appears to ignore settlement and the sampling design; consider accounting for settlement, and report at minimum a sensitivity analysis including settlement as a fixed effect.

We have done exactly this, and report the results in full. Adding settlement as a fixed effect left the estimates for sex, age, eating speed and childhood weight status essentially unchanged, attenuated the association with chronic disease (AOR 1.63, 95% CI 1.01–2.63) and removed statistical significance from the association with low household income (AOR 1.48, 95% CI 0.88–2.47). Cluster-robust standard errors treating the eight settlements as clusters left all six associations significant. Both analyses are reported in a new Results paragraph and in Supplementary Table S3, and the Discussion now states plainly that part of the between-settlement variation appears to reflect differences in the composition of the resident populations rather than the sites themselves.

3. Explain how variables were selected for the adjusted model and whether this was prespecified; report multicollinearity and justify categorisation of continuous variables such as age.

Methods now state that the covariates were specified before analysis and list them by domain (sociodemographic, clinical, behavioural); selection was not driven by univariable p values. Variance inflation factors were all below 2.6, with the highest value attaching to the age categories. Age was entered in three categories to allow for a non-linear relationship with body weight and to keep the model interpretable for use in primary care; we note that this choice sacrifices some information relative to a continuous specification.

4. Odds ratios should not be interpreted as risk ratios; consider prevalence ratios, or refer consistently to prevalence odds rather than "risk", "risk factors", "predictors" or "determinants".

We have done both. Modified Poisson regression with a robust variance estimator was fitted and the resulting prevalence ratios are reported in the Results and in Supplementary Table S3: female sex 2.26 (95% CI 1.39–3.67), age ≥55 years 1.83 (1.07–3.14), fast eating 1.52 (1.21–1.92), childhood overweight 1.47 (1.15–1.87), chronic disease 1.45 (1.07–1.95) and low household income 1.44 (1.03–2.00). Throughout the Abstract, Results, Discussion and Conclusion the words "determinants", "predictors" and "risk factors" have been replaced by statements of association, and odds ratios are described as odds rather than risk.

5. Provide 95% confidence intervals for the overall prevalence and key subgroup estimates, particularly settlement-specific prevalences; the assertion about sampling variability is too strong.

Confidence intervals (Wilson score) are now given for the overall estimate (28.8%, 95% CI 25.2–32.6), for women (35.5%, 30.9–40.3) and men (13.5%, 9.1–19.4), and for every settlement (Supplementary Table S1). Figure 4 has been redrawn to show the intervals rather than point estimates alone. The between-settlement difference has been tested formally ($\chi^2 = 29.21$, df = 7, $p < 0.001$), so we retain the statement that the sites differ, but the claim that the gap between the extremes could not represent sampling variability has been removed: Manas rests on 15 participants with an interval from 41.7% to 84.8%, and we now say that site-specific estimates based on fewer than 20 participants are indicative only.

6. The argument that the crude prevalence underestimates the burden when standardised by sex is the wrong way round; comparisons with national estimates should be revised, ideally using standardised estimates.

The Reviewer is correct and we are grateful for the correction. Because women were over-represented and had far higher prevalence, standardisation towards a balanced sex distribution lowers rather than raises the estimate. Direct standardisation to an equal sex distribution gives 24.4% (95% CI 21.1–27.9, bootstrap), which is below the national figures rather than above them. The erroneous sentence has been deleted; the Abstract, Results, Discussion and Conclusion now report the standardised estimate alongside the crude one, and the comparison with national data rests on the standardised and sex-specific values (Supplementary Table S6). We have also explained the source of the imbalance: fieldwork was conducted during the day, 62 selected individuals could not be contacted, and 51 of them were men, so non-contact was strongly patterned by sex. This is described in Methods, shown in the revised Figure 2, and acknowledged as a limitation.

7. Without pre-earthquake anthropometry or a comparison group the study cannot determine whether container residence increased obesity; environmental characteristics were not analysed; several statements go beyond the data.

We have removed the claims the Reviewer identified. The statements that the obesogenic exposure of the container city “acts on top of” prior susceptibility, that structural characteristics explain the differences between settlements, and that a particular group would obtain the “largest absolute benefit” from intervention have been rewritten as statements of association or as questions for further study, and the Abstract and Conclusion have been brought into line with the Limitations. On environmental characteristics, the questionnaire did record adequacy of food storage, access to a market, availability of an area for physical activity and household size, and we have now analysed them: none was independently associated with obesity (Supplementary Table S5). We are careful to note that these are conditions as reported by individual residents and not objective measurements of the settlement environment, which we identify as a limitation and as the priority for the multilevel work that this variation calls for.

On language, the Discussion has been revised throughout towards more neutral epidemiological wording, and rhetorical formulations have been removed.

VERSION 2 - REVIEW

**Reviewer**	**2**
**Name**	**Meneguzzo, Paolo**
**Affiliation**	**Università degli Studi di Padova, Neuroscience**
**Date**	**07-Sep-2026**
**COI**	

The authors have responded carefully to the previous comments and the revised manuscript is considerably stronger. I have only a few remaining comments.

1. Low income is still presented alongside the other five factors as an independently associated characteristic in the Abstract, Discussion and Conclusion. However, this association is not robust to adjustment for settlement (AOR 1.48, 95% CI 0.88–2.47), whereas the other principal associations are more stable. I suggest explicitly describing the income finding as sensitive to model specification rather than giving it the same evidential weight as sex, age, eating speed or childhood overweight.

2. The ROC analysis is primarily explanatory/associational, yet Figure 6 describes the model as "predicting obesity," identifies an "optimal" Youden cut-off, and states that six predictors were "retained." This is potentially misleading because all covariates were entered simultaneously rather than selected into a predictive model, and no internal or external validation of predictive performance was performed. I would either remove the Youden cut-off/prediction framing or clearly describe the AUC as apparent, in-sample discrimination. "Six independently associated variables" would also be preferable to "six retained predictors."

3. The cluster-robust sensitivity analysis should be interpreted cautiously given that there are only eight settlements. The authors report conventional cluster-robust standard errors with settlement as the clustering unit and use their stability as supporting evidence. With only eight clusters, standard sandwich variance estimates may be unreliable unless an appropriate small-sample correction is used. The authors could either apply a small-cluster correction, if feasible, or simply state that this analysis is exploratory and give greater weight to the settlement fixed-effect analysis.

4. Supplementary Table S4 notes that frequency weighting produces substantially narrower confidence intervals because it inflates the effective sample size, and explicitly states that these intervals are not design-based. If weighting is intended to account for unequal inclusion probabilities, probability/survey weights with an appropriate variance estimator would be preferable. Otherwise, I would report this analysis only as a sensitivity analysis of point estimates and avoid confidence intervals that are known not to represent the sampling uncertainty.

5. Some interpretation in the Discussion still goes beyond what was directly measured.

For example, the sex difference is linked to structural exposures such as domestic labour in the 21 m^2 units and dietary constraints, while fast eating is hypothesised to reflect overcrowding and lack of dedicated dining space. These are plausible mechanisms, but they were not tested in this study. Given the authors' otherwise careful revision toward associational language, I would frame these explicitly as hypotheses requiring further investigation rather than explanations of the observed associations.

VERSION 2 - AUTHOR RESPONSE

Reviewer 2

1. Low income is still presented alongside the other five factors as an independently associated characteristic, although the association is not robust to adjustment for settlement. It should be described as sensitive to model specification rather than given the same evidential weight.

Agreed, and corrected throughout. The Abstract now states that the income association was sensitive to model specification, and the Abstract Conclusions identify older women with chronic disease as the clearest priority group while describing the income finding as provisional. In the Discussion, the paragraph on socio-economic disadvantage now states that this was the least robust of the six findings and did not survive adjustment for settlement; the principal-findings paragraph and the implications paragraph carry the same qualification, and the Conclusion has been amended in the same way. The estimate itself is unchanged and remains in Table 5, so readers can weigh it alongside the settlement-adjusted value in Supplementary Table S3.

2. The ROC analysis is primarily explanatory, yet Figure 6 describes the model as predicting obesity, identifies an "optimal" Youden cut-off, and states that six predictors were "retained". Either remove the prediction framing or describe the AUC as apparent, in-sample discrimination.

We have removed the prediction framing entirely. Figure 6 has been redrawn without the Youden point, and its title and legend now describe apparent, in-sample discrimination; the legend states explicitly that all covariates were entered simultaneously rather than selected into a prediction model, that no internal or external validation was performed, and that no diagnostic cut-off is proposed. The same wording has been added to the Methods and to the Results, the table footnote now reads "apparent AUC", and "six retained predictors" has been replaced by "six independently associated variables" in the Strengths and limitations paragraph and elsewhere. Sensitivity and specificity values are no longer reported anywhere in the manuscript.

3. The cluster-robust sensitivity analysis should be interpreted cautiously given only eight settlements. Either apply a small-cluster correction or state that the analysis is exploratory and give greater weight to the settlement fixed-effect analysis.

We have taken the second option, which we think is the more honest one with eight clusters. The Methods now state that conventional sandwich estimators are unreliable with so few clusters, that the cluster-robust analysis is therefore reported as exploratory, and that greater weight is given to the settlement fixed-effect model. The corresponding sentence in the Results and the footnote to Supplementary Table S3 have been amended to match. We considered a wild cluster bootstrap, but with eight clusters and a binary outcome its own small-sample behaviour would be difficult to defend, so we preferred to state the limitation plainly rather than add a second uncertain estimator.

4. Supplementary Table S4 reports confidence intervals from frequency weighting that are known not to represent sampling uncertainty. Either use probability weights with an appropriate variance estimator, or report point estimates only.

Confidence intervals have been removed from Supplementary Table S4, which now reports point estimates only. The footnote explains that frequency weights inflate the effective sample size, that the resulting intervals would not represent the sampling uncertainty of the design, and that the table should be read as a sensitivity check on the point estimates alone. The corresponding sentence in the Methods has been amended in the same terms. We did not move to probability weights

because selection was made at the level of the individual with approximately equal probabilities, so there is no unequal inclusion probability for such weights to correct; the household-size analysis is retained only to show that the modest residual imbalance does not affect the conclusions.

5. Some interpretation in the Discussion still goes beyond what was directly measured, for example the structural exposures invoked for the sex difference and the overcrowding explanation for fast eating.

Both passages have been reframed as hypotheses. The sentence introducing the three structural exposures now states that none of them was measured in this study and that each remains a hypothesis for further work. The passage on eating speed now says that meal duration may plausibly be shorter because of overcrowding and the absence of a dedicated dining area, that neither was measured here, and that this remains to be tested. We have also read through the Discussion once more for similar formulations and adjusted the wording where explanation had been implied rather than demonstrated.
