# Supplementary material for: Obesity among adults living in post-earthquake container settlements: a cross-sectional study in Malatya, Türkiye
Source: BMJ Open. 2026 Sep 23;16(9):e122076. doi: 10.1136/bmjopen-2026-122076 (PMC13629913; doi:10.1136/bmjopen-2026-122076)
Supplement: Supplementary file 2 [file bmjopen-16-9-s001.docx]

**Supplementary material**

Obesity Among Adults Living in Post-Earthquake Container Settlements: A Cross-Sectional Study in Malatya, Türkiye

**Supplementary Table S1. Household-size weighted analysis**

| **Estimate** | **Unweighted** | **Weighted by household size** |
| --- | --- | --- |
| Obesity prevalence (%) | 28.8 | 27.8 |
| Female sex, OR | 3.46 | 3.62 |
| Age ≥55 y, OR | 2.44 | 2.48 |
| Fast eating, OR | 2.28 | 2.18 |
| Childhood overweight, OR | 1.93 | 1.79 |
| Chronic disease, OR | 1.85 | 2.09 |
| Low household income, OR | 1.73 | 1.68 |

*Because one adult was retained per household, adults in larger households had a marginally lower probability of appearing in the sample. Weighting each participant by the number of people living in the container unit changed the prevalence estimate by one percentage point and left the direction and magnitude of all associations unchanged. Only point estimates are reported: frequency weights inflate the effective sample size and the resulting intervals would not represent the sampling uncertainty of the design, so confidence intervals have been omitted and this table should be read as a sensitivity check on the point estimates alone.*

**Supplementary Table S2. Benjamini–Hochberg adjusted p values for the bivariate analyses in Table 4**

| **Variable** | **Unadjusted p** | **BH-adjusted p** | **Significant after adjustment** |
| --- | --- | --- | --- |
| Age group | <0.001 | <0.001 | Yes |
| Sex | <0.001 | <0.001 | Yes |
| Marital status | 0.134 | 0.229 | No |
| Education | <0.001 | <0.001 | Yes |
| Employment | <0.001 | <0.001 | Yes |
| Household income | <0.001 | <0.001 | Yes |
| Social security | 0.068 | 0.131 | No |
| Having children | 0.010 | 0.031 | Yes |
| Childhood weight status | 0.006 | 0.021 | Yes |
| Chronic disease | <0.001 | <0.001 | Yes |
| Physical injury | 0.186 | 0.283 | No |
| Adequate nutrition | 0.012 | 0.032 | Yes |
| Number of daily meals | 0.029 | 0.059 | No |
| Regular breakfast | 0.469 | 0.591 | No |
| Eating speed | 0.017 | 0.042 | Yes |
| Red meat | 0.416 | 0.549 | No |
| White meat | 0.772 | 0.829 | No |
| Vegetables | 0.019 | 0.042 | Yes |
| Pastry/dough | 0.299 | 0.433 | No |
| Dairy | 0.506 | 0.611 | No |
| Packaged food | 0.130 | 0.229 | No |
| Sugary beverages | 0.401 | 0.549 | No |
| Regular exercise | 0.863 | 0.863 | No |
| Activity area availability | 0.708 | 0.790 | No |
| Change in physical activity | 0.840 | 0.863 | No |
| Smoking | 0.011 | 0.031 | Yes |
| Alcohol use | 0.143 | 0.230 | No |
| Change in dietary habits | 0.531 | 0.616 | No |
| Effect of container living on weight | <0.001 | <0.001 | Yes |

*Twenty-nine chi-square tests were corrected using the Benjamini–Hochberg procedure at a false discovery rate of 5%. Fourteen associations were significant before correction and thirteen remained so afterwards; only the number of daily meals fell below the threshold.*

**Supplementary Table S3. Sensitivity analyses for the multivariable model**

| **Variable** | **Main model, OR (95% CI)** | **With settlement fixed effects, OR (95% CI)** | **Cluster-robust SE, OR (95% CI)** | **Modified Poisson, PR (95% CI)** |
| --- | --- | --- | --- | --- |
| Female sex | 3.46 (1.86–6.44) | 3.34 (1.76–6.33) | 3.46 (1.42–8.41) | 2.26 (1.39–3.67) |
| Age 35–54 y | 2.14 (1.14–4.03) | 2.10 (1.11–3.99) | 2.14 (1.14–4.02) | 1.75 (1.06–2.90) |
| Age ≥55 y | 2.44 (1.17–5.09) | 2.55 (1.20–5.43) | 2.44 (1.16–5.13) | 1.83 (1.07–3.14) |
| Fast eating | 2.28 (1.39–3.74) | 2.24 (1.35–3.71) | 2.28 (1.58–3.28) | 1.52 (1.21–1.92) |
| Childhood overweight | 1.93 (1.21–3.07) | 1.95 (1.21–3.15) | 1.93 (1.21–3.08) | 1.47 (1.15–1.87) |
| Chronic disease | 1.85 (1.17–2.94) | 1.63 (1.01–2.63) | 1.85 (1.20–2.87) | 1.45 (1.07–1.95) |
| Low household income | 1.73 (1.05–2.86) | 1.48 (0.88–2.47) | 1.73 (1.08–2.77) | 1.44 (1.03–2.00) |

*All models were adjusted for the same covariate set (age group, sex, education, employment, income, having children, childhood weight status, chronic disease, dietary adequacy, number of daily meals, eating speed, vegetable consumption and smoking). OR, odds ratio; PR, prevalence ratio. Cluster-robust standard errors treat the eight settlements as clusters; with only eight clusters this analysis is exploratory, since conventional sandwich estimators are unreliable with so few clusters, and the settlement fixed-effect model should carry more weight. Modified Poisson regression uses a Poisson working model with robust variance and yields prevalence ratios. Five of the seven estimates were stable across all specifications; the association with low household income lost statistical significance when settlement fixed effects were included, and the association with chronic disease was attenuated.*

**Supplementary Table S4. Obesity prevalence by container settlement, with 95% confidence intervals**

| **Settlement** | **Participants (n)** | **Obese (n)** | **Prevalence (%)** | **95% CI (%)** |
| --- | --- | --- | --- | --- |
| Yeşilyurt-Mabek | 181 | 34 | 18.8 | 13.8–25.1 |
| Yeşilyurt-İkizce | 15 | 3 | 20.0 | 7.0–45.2 |
| Yeşilyurt-MİAD | 9 | 2 | 22.2 | 6.3–54.7 |
| Yeşilyurt-Rönesans | 54 | 14 | 25.9 | 16.1–38.9 |
| Battalgazi-Malet | 125 | 37 | 29.6 | 22.3–38.1 |
| Battalgazi-TeknoKent | 114 | 39 | 34.2 | 26.1–43.3 |
| Yeşilyurt-Yeni Hayat | 50 | 23 | 46.0 | 33.0–59.6 |
| Yeşilyurt-Manas | 15 | 10 | 66.7 | 41.7–84.8 |
| All settlements | 563 | 162 | 28.8 | 25.2–32.6 |

*Confidence intervals were calculated using the Wilson score method. The difference across settlements was statistically significant (χ² = 29.21, df = 7, p < 0.001). Estimates for Manas, İkizce and MİAD rest on fewer than 20 participants each and are correspondingly imprecise.*

**Supplementary Table S5. Settlement-level environmental characteristics and obesity**

| **Characteristic** | **Obesity prevalence (%)** | **p (bivariate)** | **Adjusted OR (95% CI)** | **p (adjusted)** |
| --- | --- | --- | --- | --- |
| Adequate food storage: yes | 32.7 | 0.015 | 1 (reference) | – |
| Adequate food storage: no | 22.7 |  | 0.66 (0.42–1.03) | 0.070 |
| Access to market: easy | 26.5 | 0.131 | 1 (reference) | – |
| Access to market: difficult | 32.8 |  | 1.20 (0.77–1.87) | 0.417 |
| Activity area available: yes | 30.0 | 0.708 | 1 (reference) | – |
| Activity area available: no | 28.1 |  | 0.85 (0.55–1.33) | 0.489 |
| Household size <5 | 28.6 | 0.977 | 1 (reference) | – |
| Household size ≥5 | 29.1 |  | 1.06 (0.66–1.72) | 0.802 |
| Length of container residence <25 months | 28.1 | 0.824 | – | – |
| Length of container residence ≥25 months | 29.4 |  | – | – |

*Adjusted odds ratios are from the main multivariable model with the four environmental variables added simultaneously. None of the environmental characteristics recorded in the questionnaire was independently associated with obesity. Participants reporting adequate food storage had a somewhat higher prevalence of obesity, which most plausibly reflects household resources rather than a protective effect of limited refrigeration. These items describe conditions as reported by individual residents and are not objective measurements of the settlement environment.*

**Supplementary Table S6. Standardised prevalence estimates**

| **Estimate** | **Prevalence (%)** | **95% CI (%)** |
| --- | --- | --- |
| Crude prevalence | 28.8 | 25.2–32.6 |
| Women | 35.5 | 30.9–40.3 |
| Men | 13.5 | 9.1–19.4 |
| Standardised to an equal sex distribution | 24.4 | 21.1–27.9 |
| Household-size weighted | 27.8 | – |

*Direct standardisation was applied using the sex distribution of the Turkish adult population, which is close to 1:1. Because women were over-represented in the achieved sample and had markedly higher obesity prevalence than men, the standardised estimate is lower than the crude estimate. The confidence interval for the standardised estimate was obtained from 5000 bootstrap resamples.*
