# Supplementary material for: Obesity among adults living in post-earthquake container settlements: a cross-sectional study in Malatya, Türkiye
Source: BMJ Open. 2026 Sep 23;16(9):e122076. doi: 10.1136/bmjopen-2026-122076 (PMC13629913; doi:10.1136/bmjopen-2026-122076)
Supplement: Supplementary file 3 [file bmjopen-16-9-s002.docx]

**Supplementary File 1. Study questionnaire**

Obesity Among Adults Living in Post-Earthquake Container Settlements: A Cross-Sectional Study in Malatya, Türkiye

*The instrument was administered in Turkish by trained interviewers. The English translation below preserves the wording and the response options of the original. Items 36–38 were answered only by participants who reported that they were currently trying to lose weight (item 35).*

**Introductory statement read to participants**

This study aims to investigate the prevalence of obesity and its associated risk factors among individuals aged 18 years and over. All records kept about you in this study will remain strictly confidential. The results will not contain any name or mark identifying you. Thank you for taking part.

Settlement: 1. Teknokent 2. Malet 3. Manas 4. MİAD 5. Rönesans 6. Mabek 7. Yeni Hayat 8. İkizce

Weight ……… kg	Height ……… cm	BMI ………

**Section 1. Sociodemographic characteristics**

**1. What is your sex?**

1. Female	2. Male

**2. How old are you? ………**

**3. What is your marital status?**

1. Married 2. Single 3. Widowed 4. Divorced 5. Living apart

**4. What is your level of education?**

1. Not literate 2. Literate, no formal schooling 3. Primary school 4. Secondary school 5. High school 6. University or above

**5. What is your occupation?**

1. Civil servant 2. Private sector / worker 3. Tradesperson 4. Student 5. Retired 6. Unemployed 7. Homemaker 8. Other ………

**6. How do you perceive your socioeconomic situation?**

1. Good 2. Moderate 3. Poor

**7. Do you have social security cover (SGK, private insurance, Green Card, etc.)?**

1. Yes 2. No

**8. What is the average monthly income of your household? ………**

**9. Do you have children?**

1. Yes 2. No

**10. If yes, how many children do you have? ………**

**11. Do you have a chronic disease? (more than one may be marked)**

1. None 2. Diabetes 3. Hypertension 4. Coronary heart disease 5. COPD or other chronic lung disease 6. Chronic kidney disease 7. Rheumatic disease (e.g. arthritis, lupus) 8. Neurological disease (e.g. epilepsy) 9. Cancer (type ………) 10. Other (specify ………)

**Section 2. Earthquake exposure and container living conditions**

**12. What was the damage level of the house you were living in after the earthquake?**

1. Undamaged 2. Slightly damaged 3. Moderately damaged 4. Severely damaged 5. Collapsed

**13. Did you sustain a physical injury during the earthquake?**

1. No injury 2. Yes, minor injury (superficial cuts, abrasions, soft-tissue trauma, bruising) 3. Yes, moderate injury (fractures, deep cuts, ligament injury) 4. Yes, serious injury (multiple trauma, internal organ damage, head or spinal injury) 5. Yes, limb loss (amputation)

**14. For how long have you been living in the container settlement? ……… months**

**15. How often do you stay in the container settlement?**

1. I live here permanently 2. I mostly live here and occasionally stay elsewhere 3. I rarely stay here and usually live elsewhere

**16. How many people in total live in your container unit? ………**

**17. Are the food storage conditions in your container adequate (e.g. a refrigerator)?**

1. Yes 2. No

**18. How would you describe your access to a shop or market?**

1. Very easy 2. Easy 3. Difficult 4. Very difficult

**19. Compared with the period before the earthquake, how have your eating habits changed in the container?**

1. Became healthier 2. Stayed the same 3. Became less healthy

**20. How has living in the container affected your weight control?**

1. Caused me to gain weight 2. Caused me to lose weight 3. Had no effect

**21. If you gained weight in the container, what do you think was the main reason?**

1. Insufficient physical activity 2. Stress and psychological circumstances 3. Unhealthy eating 4. Health problems 5. Medication use (e.g. corticosteroids) 6. I did not gain weight 7. Other (specify ………)

**22. What is the greatest difficulty you face when cooking in the container?**

1. Insufficient space 2. Lack of equipment (pots, cooker, etc.) 3. Hygiene conditions 4. Power or water cuts 5. Other (specify ………)

**23. Do you think living conditions in the container have had adverse effects on your health?**

1. Yes 2. No

**24. Have communal food arrangements in the settlement (aid distribution, canteen, etc.) affected your eating habits?**

1. No effect 2. Yes, a positive effect 3. Yes, an adverse effect

**Section 3. Physical activity and substance use**

**25. Do you exercise regularly? (regular physical activity is defined as at least three days a week and at least half an hour a day of cycling, swimming, running, walking, etc.)**

1. Yes 2. No

**26. Are there areas in the settlement where you can be physically active, and do you use them?**

1. Yes, there are and I use them 2. Yes, there are but I do not use them 3. No, there are none

**27. Has living in the container changed your physical activity habits?**

1. Yes, I used to be regularly active but can no longer be 2. Yes, I have become more active 3. I was not active before and have now started 4. I was not active before and am still not active

**28. If you do not exercise regularly, what is the reason?**

1. Health problems (e.g. musculoskeletal problems, chronic disease) 2. Lack of suitable space or equipment 3. Lack of time (work, daily responsibilities) 4. Lack of motivation or psychological reasons 5. Safety concerns 6. Financial constraints (gym, equipment) 7. I do exercise regularly

**29. Do you smoke?**

1. Yes, at least one cigarette every day 2. Occasionally, but not every day 3. I used to smoke and have quit 4. I have never smoked

**30. Do you drink alcohol?**

1. Yes, every day 2. Yes, every other day 3. Yes, once a week 4. Yes, once a month 5. Yes, on special occasions 6. No

**Section 4. Body perception, weight history and dietary habits**

**31. What do you think about your body and appearance?**

1. Underweight / thin 2. Normal weight 3. Overweight 4. Obese

**32. What was your weight like in childhood?**

1. Normal 2. Slightly overweight 3. Overweight 4. Underweight

**33. Have you ever been on a diet in your life?**

1. Yes 2. No

**34. Have you ever been diagnosed with obesity by a health professional?**

1. Yes 2. No

**35. Are you currently following a diet or any other practice to lose weight?**

1. Yes, I am 2. No, my weight is fine 3. No, but I need to lose some weight 4. No, I need to gain weight

**36. If yes, which approach are you following?**

1. Diet 2. Physical activity 3. Diet plus physical activity 4. Other (specify ………)

**37. If yes, from whom did you obtain the diet or other approach?**

1. Dietitian 2. Physician 3. Books / internet / celebrities 4. Family / friends 5. Health personnel (nurse, physiotherapist, etc.) 6. By myself (personal experience, trial and error) 7. Fitness trainer 8. Other (specify ………)

**38. If yes, what happened to your weight?**

1. I lost weight 2. I gained weight 3. My weight did not change 4. It did not work; weight control was not achieved 5. I am finding it hard but making progress

**39. In your own view, do you eat an adequate and balanced diet?**

1. Yes 2. No

**40. How many meals a day do you eat?**

1. One meal 2. Two meals 3. Three meals 4. Four or more

**41. Do you ever skip main meals?**

1. Yes 2. No 3. Sometimes

**42. If yes or sometimes, which main meal do you usually skip?**

1. Breakfast 2. Lunch 3. Dinner 4. I do not skip meals

**43. Do you eat breakfast regularly?**

1. Yes 2. No

**44. Do you eat snacks?**

1. Yes 2. No

**45. If yes, which snack do you prefer most?**

1. Before breakfast 2. Between breakfast and lunch 3. Between lunch and dinner 4. After dinner

**46. Do you eat outside the home very often?**

1. Yes 2. No

**47. How would you describe the speed at which you eat your meals?**

1. I eat fast 2. I eat at a normal speed 3. I eat slowly

**48. Which cooking method do you use most often?**

1. Frying 2. Sautéing 3. Boiling 4. Baking / oven

**49. How much water do you drink on an average day?**

1. 0–1 litre 2. 1–2 litres 3. 2–3 litres 4. 3 litres or more

**50. How many portions of bread do you consume on an average day? ………**

**51. Which type of fat do you most often use in cooking?**

1. Olive oil 2. Other vegetable oils (sunflower / corn) 3. Butter 4. Margarine 5. Animal fats (tail fat, suet)

**52. How often do you consume the following food groups? (mark one option for each item with an X: daily / 1–2 times a week / 3 or more times a week / rarely / never)**

Red meat (e.g. beef, lamb) · White meat (e.g. chicken, fish) · Pulses (e.g. lentils, chickpeas) · Vegetables (e.g. leafy greens, root vegetables) · Pastry and dough-based foods (e.g. börek, pizza) · Milk and dairy products (e.g. milk, yoghurt, cheese) · Fruit · Ready-to-eat foods (e.g. packaged snacks, fast food) · Sugar-sweetened beverages (e.g. cola, fruit juice) · Salad eaten with meals

*Note. Seven of the ten food-frequency items (red meat, white meat, vegetables, pastry and dough-based foods, dairy products, ready-to-eat foods and sugar-sweetened beverages) were used in the analyses reported in the manuscript. For analysis, responses were dichotomised as consumption three or more times per week versus less often.*
